# A visual perception-guided framework for camera path planning in large-scale digital twin and AI-generated 3D scenes

**DOI:** 10.1371/journal.pone.0346542

**Published:** 2026-09-17

**Authors:** Yan Zhang, Wei Chen, Gang Yang

**Affiliations:** 1 Arts and Media School, Century College, Beijing University of Posts and Telecommunications, Beijing, China; 2 Arts and Media School, Century College, Beijing University of Posts and Telecommunications, Beijing, China; 3 School of Information Science & Technology, Beijing Forestry University, Beijing, China; Universitas Multimedia Nusantara, INDONESIA

## Abstract

This study investigates visual, cognitive, and spatial saliency indicators of architectural landmarks in large-scale ancient city ruins and digital twin virtual environments. It further examines users’ cognitive demands during pathfinding in large, complex environments and explores how different exploration strategies interact with interact with architectural spatial layouts. We propose a virtual camera exploration method guided by visual perception principles. Comparative experiments with several representative path optimization algorithms show that the proposed method outperforms existing methods and provides effective optimization strategies. This method identifies visually engaging and aesthetically rich regions in large-scale, complex virtual environments. As a result, it improves users’ exploration efficiency in such environments with multiple layers and complex structures, while enhancing their scene understanding and aesthetic experience. The proposed method has substantial practical value across multiple fields, including landscape animation, 3D heritage simulation, and virtual cultural tourism.

## 1. Introduction

Path planning for virtual cameras is a crucial technique for understanding virtual reality scenes and has been widely applied in fields such as *medical visualization*, *virtual simulation*, *field robotics* [1], *cultural heritage preservation*, *virtual scenic navigation, mechanical simulation* [2], *point cloud processing* [3], and *interactive gaming*. This study introduces a virtual camera path planning method guided by visual perception principles. The proposed method enables intelligent roaming and exploration in large-scale, complex virtual environments, with the aim of maximizing the acquisition of aesthetically relevant scene features and visually engaging information. Specifically, the method guides the virtual camera toward informative viewpoints of 3D objects to explore visually rich areas. Furthermore, it integrates an ant colony algorithm with a *dynamic artificial potential field* (DAPF-based) force model to enable efficient path planning for the virtual camera. The proposed framework is initially validated on digital twin scenes of ancient heritage parks, and subsequently extended to natural landforms, digital cities and text-to-3D generated scenarios to evaluate its generalization performance. Comparative experimental results indicate that the proposed method performs satisfactorily under the tested conditions.

This methodology is particularly well-suited for the exploration of large, intricate 3D environments, especially for capturing visually salient areas and aesthetically meaningful scene information. It has practical implications for digital twin path planning, virtual camera navigation guidance, hierarchical scene understanding, and the analysis of visually engaging regions. This framework unifies path planning for traditional heritage digital twin data and AIGC-based Text-to-3D generated scenes, solving the visual guidance problem of virtual camera roaming in both real reconstructed scenes and AI-created complex environments.

## 2. Human eye attention mechanism in virtual reality for path planning

The visual appeal of landscape nodes in virtual scenes is characterized by distinct visual features, reflecting interactions between the observer and the overall virtual environment during *wayfinding*. Previous studies have shown that object saliency is closely associated with regions of interest during *wayfinding* observations [4]. The visual saliency features of scene objects are widely recognized as critical factors in attracting wayfinders’ attention during landmark identification. These visually engaging attributes enable wayfinders to more effectively perceive and distinguish spatial objects that exhibit significant contrasts with their surrounding environments during *perceptual navigation* [5], thereby facilitating active exploration of landmark buildings or landscape nodes in virtual scenes.

In practical and commercial applications, virtual camera control based on optimal viewpoint sets has driven the development of numerous products and patents. Related research has also produced multiple patented innovations [6]. For example, Kahng et al.’s patent introduces a multi-viewpoint virtual reality system aimed at enhancing scene understanding [7]. Piemonte et al.’s patented product enables multi-directional virtual cameras for visualizing 3D maps [8], whereas Vandrotti et al.’s patent employs a multi-camera virtual reality system that automatically adjusts scene navigation [9]. These products demonstrate the critical role of virtual camera path planning in enhancing scene understanding across various practical applications. This paper proposes a mesh *saliency-enhanced* approach to identify visually salient regions in large-scale, complex scenes, thereby enabling intelligent camera path planning.

### 2.1. Evaluation of scene object visibility

Current research on 3D mesh saliency detection focuses on three main methods: Gaussian curvature-based detection [10], *vertex uniqueness analysis* [11], and *region of interest* (ROI) computation [12], all based on the principles of *Gestalt psychology* [13]. Notably, the *curvature-weighted* method [10] has been used to enhance scene understanding through perceptual modeling and computer vision architectures [14].

Saliency analysis is based on Itti et al.’s *center-surround mechanism* [15], later extended to 3D using Lee’s *MeshSaliency* algorithm with *Gaussian curvature weighting* [10]. This framework demonstrates the effectiveness of curvature entropy in modeling visual attention during geometric processing [16], [17], offering a neurobiologically inspired approach for feature integration.

Recent advances in mesh ROI analysis include Leifman’s *curvature-based method* [11] and Bartolomeo’s *neurocognitive model* [18]. Kim et al. validated these methods, demonstrating a strong correlation between the *curvature-weighted model* and human gaze patterns [19], thereby confirming their usefulness in mesh simplification.

Viewpoint selection research has yielded several optimization techniques—including 2D-3D classifiers [20], linear prediction models [12], and image voting methods [21]—all intended to improve user experience in virtual environments. Automatic diffusion functions employ *Laplace-Beltrami* eigenfunction analysis and *3D Harris curvature* detection to identify mesh protrusions. Recent advances include graph optimization, *f-divergence* methods, and machine learning solutions [22–24], which collectively improve 3D geometric analysis.

This paper investigates the implicit effects of visual perception on user behavior, making the identification of visually engaging regions within a scene a crucial step [10]. Directing the virtual camera to regions rich in visual information presents a major challenge. In this study, an approach is proposed to guide the camera’s orientation based on the information richness at its viewpoint, thereby highlighting regions with abundant visual content.

*Gaussian weighting* is employed to identify the visually distinctive value $J\left(v_{i},\sigma \right)$ across multiple scales, as defined in Equation (1), in which $Q\left(v_{i},\sigma \right)$ denotes the mean Gaussian curvature of the vertex within a σ-scale range:


$$\begin{array}{c} J\left(v_{i},\sigma \right)=\frac{\sum_{v\in N\left(v_{i},2\sigma \right)}Q\left(v_{i},\sigma \right)}{\sum_{v\in N\left(v_{i},2\sigma \right)}exp[-||v-v_{i}|{|}^{\text{2}}/\left(2\sigma^{\text{2}}\right)]} \end{array}$$ (1)

Subsequently, the visual uniqueness of the vertex $v_{i}$ at scale σ is given by Equation (2):


$$\begin{array}{c} \vartheta \left(v_{i}\right){|}_{\sigma }=\left|J\left(v_{i},\sigma )-J(v_{i},2\sigma \right)\right| \end{array}$$ (2)

Considering a total of j scales, the final *grid-level* object interest value is given by Equation (3):


$$\begin{array}{c} \vartheta \left(v_{i}\right)=\sum_{k=1}^{j}S\left(\vartheta \left(v_{i}\right){|}_{\sigma_{k}}\right) \end{array}$$ (3)

Here, S denotes the *nonlinear normalization* function proposed by Lee [22]. Using this method, we derive the visual interest degree ϑ(O) of the grid. Q serves as a metric for assessing visual interest. Notably, the visibility of an object grid depends on the camera’s viewpoint; if an object is occluded, the region of interest cannot be observed. Therefore, we adopt the following strategy to achieve this goal.

- *Visibility weight formulation for Patch-Level evaluation*

To quantify the visibility of object patches from a given camera viewpoint, we introduce a visibility weight $\omega_{vis}\left(v_{i},X_{c}\right)$. This weight reflects whether a vertex $v_{i}$ on an object patch contributes to the visible region from the camera’s current position $X_{c}$, as formalized in Equation (4):


$$\begin{array}{c} \omega_{vis}\left(v_{i},X_{c}\right)=\left\{ \begin{array}{c} 1,\text{if}\;cos\frac{\alpha }{\text{2}}\leq \frac{s\cdot d\left(v_{i},X_{c}\right)}{∥\cdot ∥v_{i}-X_{c}∥}\text{and}\;v_{i}\in V\left(P_{\text{0}}^{k}\right),\\ \text{0,otherwise.} \end{array}\right. \end{array}$$ (4)

In this formulation, *s* denotes the *line-of-sight* direction of the camera viewpoint $X_{c}$, defining the orientation along which visibility is evaluated; $d\left(v_{i},X_{c}\right)$represents the Euclidean distance from $X_{c}$ to vertex $v_{i}$ (belonging to object *O*) along the direction *s*; $V\left(P_{\text{0}}^{k}\right)$ is the set of vertices composing the *k-th* object patch, ensuring the weight is computed at the patch level for localized visibility analysis; and the term $cos\frac{\alpha }{\text{2}}\leq \frac{s\cdot d\left(v_{i},X_{c}\right)}{∥\cdot ∥v_{i}-X_{c}∥}$ quantifies the angular condition for visibility, determining whether $v_{i}$ lies within the camera’s view frustum by comparing the cosine of half the view angle *α* with the normalized dot product of *s* and the vector from $X_{c}$ to $v_{i}$.

- *Overall visibility evaluation of scene objects*

To provide a comprehensive evaluation of the visibility of an object *O* from a camera viewpoint $X_{c}$, we propose a multi-step framework integrating *patch-level* visibility, spatial extent, and visual interest. The formulation is structured as follows:

*Step 1: Patch-Level visibility aggregation*

First, we compute the *patch-level* visibility sum $S_{patch}$, which aggregates the visibility contributions of all vertices across the object’s patches:


$$\begin{array}{c} S_{patch}=\sum_{i=1}^{n_{p}}\frac{\omega_{vis}\left(v_{i},X_{c}\right)}{d\left(v_{i},X_{c}\right)} \end{array}$$ (5)

Here, $n_{p}$ denotes the number of patches in *O*. The term $\omega_{vis}\left(v_{i},X_{c}\right)$ is the visibility weight of vertex $v_{i}$ (defined in Equation (4)), and $d\left(v_{i},X_{c}\right)$ is the distance from $X_{c}$to $v_{i}$. This summation normalizes vertex visibility by distance, ensuring a spatially balanced aggregation.

*Step 2: Scene object bounding box (spatial extent)*

Next, we characterize the normalized maximum bounding box S(O) of *O*, capturing the object’s spatial scale in 3D space:


$$\begin{array}{c} S\left(O\right)=S_{Normal}\left(\sum_{dim\in \left\{x,y,z\right\}}{max}_{o\in O}|o_{max}-o_{min}|\right) \end{array}$$ (6)

In this equation, $S_{Normal}$ is a normalization function standardizing the bounding box across different objects. The summation over *x*, *y*, and *z* dimensions computes the total spatial extent, with ${max}_{o\in O}|o_{max}-o_{min}|$ representing the maximum extent of *O* along each axis.

*Step 3: Overall visibility integration*

Finally, the overall visibility $V\left(X_{c},O\right)$ of *O* from $X_{c}$ is derived by combining patch-level visibility, spatial extent, and visual interest:


$$\begin{array}{c} V\left(X_{c},O\right)=\frac{C\left(S_{patch}\right)}{C\left(O\right)}\cdot \left(S\left(O\right)+\vartheta \left(O\right)\right) \end{array}$$ (7)

Here, *C*(*O*) is the total number of patches in *O*, ensuring $\frac{C\left(S_{patch}\right)}{C\left(O\right)}$ normalizes the patch-level sum. ϑ(O) denotes the normalized visual interest value of *O* (from Equation (3)), integrating the object’s semantic importance into the visibility assessment.

- *Visual information gain for next-viewpoint selection*

To quantify the new visual information introduced by a candidate next viewpoint $X_{next}$, we define the visual information gain $G\left(X_{next}\right)$. This metric integrates the visibility of the next object, accumulated trajectory information, and visual interest, as formulated below:


$$\begin{array}{c} G_{v}(X_{next})=\frac{V(X_{next},O_{next})\cdot (V(X_{next},O_{next})⋂\Phi \left(X_{o}^{k}\right))\left|V\left(X_{c}\right)\right|\cdot \vartheta \left(O_{next}\right)}{\left|V\left(X_{c}\right)\right|} \end{array}$$ (8)

In this formulation, the visibility gain integrates several key components. The term $V\left(X_{next},O_{next}\right)$ represents the visibility of the object $O_{next}$ from the candidate viewpoint $X_{next}$, reflecting the portion of $O_{next}$ that is observable. $\Phi \left(X_{\text{0}}^{k}\right)$ denotes the cumulative visibility information along the traversed camera trajectory $X_{\text{0}}^{k}=\{X_{\text{0}},X_{\text{1}},...,X_{k}\}$, ensuring that we account for previously observed scene content. $\left|V\left(X_{c}\right)\right|$ indicates the visibility magnitude at the current viewpoint $X_{c}$, normalizing the gain to maintain comparability across different current positions. $\vartheta \left(O_{next}\right)$ signifies the visual interest of $O_{next}$, weighting the gain by the object’s semantic and perceptual importance (derived from Equation (3)).

The term $V\left(X_{next},O_{next}\right)⋂\Phi \left(X_{\text{0}}^{k}\right)$ measures the overlap between newly observed visibility and previously accumulated information, ensuring that viewpoints introducing truly novel content are prioritized, thereby enhancing exploration efficiency.

- *Viewpoint selection heuristic function*

To rank candidate next viewpoints and identify the optimal one, we introduce the viewpoint selection heuristic function $E_{v}\left(X_{c}\right)$. This function balances visual information gain, object interest, and spatial proximity to *high-priority* objects, as defined in Equation (9):


$$\begin{array}{c} E_{v}\left(X_{c}\right)=\frac{d\left(X_{c},X_{g}\right)\cdot \left(G_{v}\left(X_{next})+\vartheta (O_{x_{g}}\right)\right)}{d\left(X_{next},X_{g}\right)} \end{array}$$ (9)

In this formulation, $X_{g}$ denotes the position of objects ranked in the top 10 for visual interest (identified via the *Top-10 method*), prioritizing regions with high semantic and perceptual value; $O_{x_{g}}$ is the target object located at $X_{g}$, serving as a key focus for viewpoint navigation; d(·,·) represents the geodesic distance between camera positions, capturing the spatial cost of moving between viewpoints; $G_{v}\left(X_{next}\right)$ is the visual information gain at the candidate next viewpoint $X_{next}$ (derived from Equation (7)), quantifying the novelty of visual content; $\vartheta \left(O_{x_{g}}\right)$ signifies the visual interest of $O_{x_{g}}$, weighting the heuristic by the object’s importance.

The numerator $d\left(X_{c},X_{g}\right)\cdot \left(G_{v}\left(X_{next})+\vartheta (O_{x_{g}}\right)\right)$ integrates the camera’s distance to $X_{g}$ with the combined visual gain and object interest, while the denominator $d\left(X_{next},X_{g}\right)$ normalizes this by the next viewpoint’s distance to $X_{g}$, ensuring the heuristic favors viewpoints that are both information-rich and spatially efficient. Through these strategies, we successfully incorporate visual information into the guidance of camera movement, paving the way for the design of an intelligent path planning method.

### 2.2. Improved DPC-based potential field force algorithm

The artificial potential field method, proposed by Khatib et al. [25], is widely adopted in robotics. It guides an agent toward a target via potential field functions: the target exerts an attractive force, while obstacles generate repulsive forces. The net force dictates the agent’s motion. However, this method has inherent limitations. When the target is distant, strong attraction may lead to collisions if it dominates repulsion. As the agent nears the target, attraction weakens; nearby obstacles may then exert strong repulsion, potentially impeding arrival at the target. Moreover, if attraction and repulsion balance precisely, the agent may stall or oscillate. To overcome these issues, we introduce the following strategies.

- *Attractive potential energy formulation*

Let $X=[x,y{]}^{\text{T}}$ denote a grid point’s position, $X_{c}$ represent the camera’s current position, and $X_{g}$ denote the target point’s location. If $d\left(X_{c},X_{g}\right)\leq d_{goal}^{*}$, the gravitational force is given by Equation (10):


$$\begin{array}{c} U_{att}(X_{c})=\frac{\text{1}}{\text{2}}k_{att}(d(X_{c},X_{g}){)}^{\text{2}} \end{array}$$ (10)

If $d\left(X_{c},X_{g}\right)>d_{goal}^{*}$, Equation (11) applies:


$$\begin{array}{c} U_{att}\left(X_{c}\right)=k_{att}d_{goal}^{*}d\left(X_{c},X_{g}\right)-\frac{\text{1}}{\text{2}}(d_{goal}^{*}{)}^{\text{2}} \end{array}$$ (11)

Here, $k_{att}$ denotes the gravitational potential field constant, $d\left(X_{c},X_{g}\right)$ denotes the distance between the current position $X_{c}$ of the agent and the target point $X_{g}$, while $d_{goal}^{*}$ signifies the distance threshold.

If $d\left(X_{c},X_{g}\right)\leq d_{goal}^{*}$, the gravitational force equals the potential field gradient, given by Equation (12):


$$\begin{array}{c} \nabla U_{att}\left(X_{c}\right)=k_{att}(X_{c}-X_{g}) \end{array}$$ (12)

If $d\left(X_{c},X_{g}\right)>d_{goal}^{*}$, the gravitational force is calculated via Equation (13):


$$\begin{array}{c} \nabla U_{att}\left(X_{c}\right)=k_{att}d_{goal}^{*}\frac{\left(X_{c}-X_{g}\right)}{d\left(X_{c},X_{g}\right)} \end{array}$$ (13)

If obstacles exist near the target point, the agent may become unreachable. Accordingly, the strategy is further refined as described below.

- *Repulsive Potential Energy Formulation*

To ensure collision avoidance while maintaining *goal-oriented* navigation, we define the repulsive potential energy $U_{rep}\left(X_{c}\right)$ for scenarios where the camera’s current position $X_{c}$ lies within the influence radius $d_{\text{0}}$ of an obstacle $X_{o}$. This formulation is given by Equation (14):


$$\begin{array}{c} U_{rep}\left(X_{c}\right)=\left\{ \begin{array}{c} \frac{\text{1}}{\text{2}}k_{rep}\cdot T_{obs}\cdot d^{n}\left(X_{c},X_{g}\right),ifd\left(X_{c},X_{o}\right)\leq d_{\text{0}},\\ 0,\text{otherwise} \end{array}\right. \end{array}$$ (14)

Here, $T_{obs}$ is defined by Equation (15):


$$\begin{array}{c} T_{obs}={\left(\frac{\text{1}}{d\left(X_{c},X_{o}\right)}-\frac{\text{1}}{x_{\text{0}}}\right)}^{\text{2}} \end{array}$$ (15)

In this formulation, $k_{rep}$ is a positive constant controlling the intensity of the repulsive force, with larger values enabling more aggressive obstacle avoidance; $d\left(X_{c},X_{o}\right)$ denotes the Euclidean distance between $X_{c}$ and the obstacle $X_{o}$, while$d_{\text{0}}$ represents the maximum radius within which the obstacle exerts a repulsive effect; $x_{\text{0}}$ serves as a reference distance to prevent numerical singularities when $X_{c}$ is very close to $X_{o}$; and *n*, typically set to 2, is a positive integer that weights the contribution of $d\left(X_{c},X_{g}\right)$ (the distance from $X_{c}$ to the target $X_{g}$), ensuring the repulsive force diminishes as the camera approaches its goal.

- *Repulsive force for sub-scene areas*

In this formulation, the repulsive force $F_{rep_{\text{1}}}\left(X_{c}\right)$ exerted on an entity at position $X_{c}$ acts when its distance $d\left(X_{c},X_{o}\right)$ from a sub-scene’s reference point $X_{o}$ falls within the range $R_{o}<d\left(X_{c},X_{o}\right)<R_{o}+\sigma_{o}$.

To define $F_{rep_{\text{1}}}\left(X_{c}\right)$, we introduce the following components in a concise description: $Frep_{\text{2}}\left(X_{c}\right)$ represents the base repulsive force arising from sub-scene interactions; $d\left(X_{c},x_{g}\right)$ denotes the *Euclidean distance* between the entity’s position $X_{c}$ and a target $x_{g}$; $R_{o}$ is the radial distance from the sub-scene’s center node to the edge of its region; and $\sigma_{o}$ specifies a safety distance buffer near sub-scenes, ensuring minimal interference margins.

*Step 1: Exponential attenuation term*

The exponential attenuation term Atten modulates the base repulsive force according to spatial distance, reflecting how the force gradually diminishes with distance. It is computed as:


$$\begin{array}{c} Atten=e^{-\frac{d\left(X_{c},x_{g}\right)2}{2(R_{o}+\sigma_{o}{)}^{\text{2}}}} \end{array}$$ (16)

*Step 2: Repulsive force calculation*

The final repulsive force $F_{rep_{\text{1}}}\left(X_{c}\right)$ is obtained by scaling the base repulsive force$F_{rep_{\text{2}}}\left(X_{c}\right)$ with the attenuation term:


$$\begin{array}{c} F_{rep1}\left(X_{c}\right)=F_{rep2}\left(X_{c}\right)\cdot Atten \end{array}$$ (17)

Thus, we achieve the improved repulsive force computation.

- *Enhanced repulsive force for sub-scene areas*

This formula defines the enhanced repulsive force $F_{rep_{\text{2}}}\left(X_{c}\right)$ acting on an entity at position $X_{c}$ when its distance $d\left(X_{c},X_{o}\right)$ from a sub-scene’s reference point $X_{o}$ lies within $R_{o}+\sigma_{o}\leq d\left(X_{c},X_{o}\right)<R_{o}+\sigma_{o}+d_{o}$. Key components and variables used in this formulation are defined as follows:

Parameters $k_{rep}$, $d\left(X_{c},X_{o}\right)$, $d_{\text{0}}$, $d\left(X_{c},X_{g}\right)$, are consistent with those in Equation (13); The unit vector ${\vec{n}}_{oc}$ points from the obstacle to the entity’s position; the safety distance $\sigma_{o}$ defines the minimum clearance near sub-scenes; the radius represents an extension of the obstacle’s influence range, governing the range of repulsive effects; and the vector ${\vec{n}}_{cg}$ points from the entity’s position to the target.

*Component 1: Obstacle-induced repulsive term*

This term captures the repulsive effect directly induced by obstacles. It is formulated as:


$$\begin{array}{c} T_{\text{1}}=k_{rep}\left(\frac{\text{1}}{d\left(X_{c},X_{o}\right)}-\frac{\text{1}}{d_{o}}\right)\cdot \frac{d^{n}\left(X_{c},X_{g}\right)}{d^{n}\left(X_{c},X_{o}\right)}{\vec{n}}_{oc} \end{array}$$ (18)

*Component 2: Safety-distance-induced repulsive term*

This term accounts for repulsive forces arising from the need to maintain the *safety distance* $\sigma_{o}$. It is given by:


$$\begin{array}{c} T_{\text{2}}=\frac{n}{\text{2}}k_{rep}{\left(\frac{\text{1}}{d\left(X_{c},X_{o}\right)}-\frac{\text{1}}{\sigma_{o}+d_{o}}\right)}^{\text{2}}d^{n-1}\left(Xc,Xg\right){\vec{n}}_{cg} \end{array}$$ (19)

*Component 3: Combined enhanced repulsive force*

The enhanced repulsive force $F_{rep_{\text{2}}}\left(X_{c}\right)$ is the vector sum of the two components above:


$$\begin{array}{c} F_{rep_{\text{2}}}\left(X_{c}\right)=T_{\text{1}}+T_{\text{2}} \end{array}$$ (20)

- *Resolution of deadlock problem*

To assess local minima conditions, equilibrium is achieved when the resultant force from attractive and repulsive forces is balanced. Additionally, if the camera exhibits little displacement over time or remains stationary, a “*deadlock*” situation may arise, which should be avoided. Inspired by Sokolov et al. [26], an additional attractive force $F_{att_{ex}}$ is introduced in this study. Equation (9) determines the direction $E_{v}\left(X_{c}\right)$ toward visually interesting areas. The resultant force direction prior to *deadlock* is denoted as $F_{tot}^{{\;}^{\prime }}$. The angle $\theta_{FE}$ between $X_{next}$ (the next position determined by $E_{v}\left(X_{c}\right)$) and the potential field’s resultant force direction is calculated as follows. Consequently, the additional attractive force is given by Equation (21):


$$\begin{array}{c} F_{att_{ex}}=\left|F_{tot}^{{\;}^{\prime }}\right|\cdot cos\theta_{FE} \end{array}$$ (21)

If $\left|F_{tot}\leq \varepsilon |⋃|S_{\Delta t}\leq S_{min}\right|$, the resultant potential field force is given by Equation (22):


$$\begin{array}{c} F_{tot}\left(X_{c}\right)=F_{att}\left(X_{c}\right)+F_{rep}\left(X_{c}\right)+F_{att_{ex}} \end{array}$$ (22)

Otherwise:


$$\begin{array}{c} F_{tot}\left(X_{c}\right)=F_{att}\left(X_{c}\right)+F_{rep}\left(X_{c}\right) \end{array}$$ (23)

Among these, $S_{\Delta t}$ denotes the *deadlock* condition threshold (displacement within Δt), and $S_{min}$ represents the critical displacement value. Building upon the visually guided enhanced potential field method, the subsequent section integrates the ant colony algorithm to refine the path selection *state transition matrix*, facilitating path optimization.

### 2.3. Potential field ant colony path planning based on scene visual uniqueness regions

This study addresses the challenge of managing virtual scenes with numerous 3D building models. Initially, it is essential to compute the visual interest degree of 3D objects in the scene. Next, we calculate optimal viewpoints for traversing building models to determine the virtual camera’s position and orientation for each. At each time step, the camera configuration parameters are stored in matrices: a position matrix records the camera’s position, and a rotation matrix captures its orientation.

A tabu list $tabu_{k}$ is established to record all reachable points during camera path planning; thus, nodes in $tabu_{k}$ cannot be revisited. The potential positions selectable by an ant at each step are denoted as $allowed_{k}=\{C-tabu_{k}\}$. The state transition probability ($j\in allowed_{k}$, $g\in goal_{k}$) is given by Equation (24):


$$\begin{array}{c} p_{ij}^{k}\left(t\right)=\frac{\tau_{ij}^{\alpha }\left(t\right)\eta_{ig}^{\beta }\left(t\right)}{\sum_{\begin{array}{c} i\in allowed_{k}\\ g\in goal_{k} \end{array}}\tau_{ij}^{\alpha }\left(t\right)\eta_{ig}^{\beta }\left(t\right)} \end{array}$$ (24)

Otherwise, $p_{ij}^{k}\left(t\right)=0$. Among these, $\tau_{ij}\left(t\right)$ represents the pheromone concentration on path (i,j) at time t. α is the *pheromone heuristic factor*, reflecting the influence of accumulated pheromones on ant movement. β is the *visibility heuristic factor*, indicating how $\eta_{ig}\left(t\right)$ affects the ant colony’s search behavior and path selection. $\eta_{ig}\left(t\right)$ refers to the *heuristic expectation* between reachable points i and g from the camera position at time t; in standard ant colony algorithm implementations, it is typically $\eta_{ig}\left(t\right)=1/d_{ig}\left(t\right)$.

Under optimal viewpoint guidance, selecting the heuristic function $\eta_{ig}\left(t\right)$ requires integrating potential field force information and the viewpoint selection heuristic function. Here, vector ${\vec{F}}_{E_{v}}$ is defined as the difference between the current and selected *next-best viewpoint*, and ${\vec{F}}_{tot}$ denotes the potential field force. The minimum angle $\theta_{F}$ is defined as the angle between ${\vec{F}}_{tot}$ (potential field force direction) and the direction of selectable grids. Additionally, $j=\left\lfloor \frac{8\theta_{F}+\pi }{2\pi }+1\right\rfloor$ denotes the sequence number of adjacent grids aligned with pheromone diffusion directions. Considering the influence of potential field forces, the *heuristic factor*$\eta_{ij}\left(t\right)$ must be corrected accordingly. Consequently, Equations (25) and (26) illustrate how ${\vec{F}}_{tot}$ (*path heuristic factor*) is modified by potential field forces:


$$\begin{array}{c} \eta_{ij}\left(t\right)=\frac{T_{m}-T_{c}}{T_{m}}\cdot \frac{a^{F_{tot}\cdot cos\theta_{F}}}{H_{ij}} \end{array}$$ (25)

$$\begin{array}{c} H_{ij}=d\left(X_{i},X_{j}\right)e^{-\frac{∥X_{i}-X_{j}|{|}^{\text{2}}}{2\sigma^{\text{2}}}} \end{array}$$ (26)

Among these, $j=\left\lfloor \frac{8\theta +\pi }{2\pi }+1\right\rfloor$, $d\left(X_{i},X_{j}\right)$ denotes the distance between $X_{i}$ and $X_{j}$. $\theta_{F}$ represents the smallest angle between ${\vec{F}}_{tot}$ and available adjacent grids. σindicates the sub-scene group neighborhood scale in the *K-opt* algorithm.

Let ${\vec{O}}_{E_{v}}$ denote the direction with the highest $E_{v}\left(p\right)$ (visual interest guidance value) among the available directions at the ant’s current position $p_{c}$. The angle between ${\vec{F}}_{tot}$, ${\vec{O}}_{E_{v}}$, and $p_{c}$ is $\theta_{E}$. If unobstructed directions exist at $p_{c}$, the probability of selecting the grid corresponding to the minimum $\theta_{FE}$ (between ${\vec{F}}_{tot}$ and ${\vec{O}}_{E_{v}}$) is given by Equation (27):


$$\begin{array}{c} \delta_{ij}\left(t\right)=\left\{ \begin{array}{c} 1,\begin{array}{c} \;if\;cos\theta_{FE}\subset \left\lfloor (j-1)\times \frac{\pi }{\text{4}}-\frac{\pi }{\text{8}},(j-1)\times \frac{\pi }{\text{4}}+\frac{\pi }{\text{8}}\right\rfloor \end{array}\\ 0.5,\begin{array}{c} \;if\;cos\theta_{FE}\subset \left\lfloor (j-1)\times \frac{\pi }{\text{4}}+\frac{\pi }{\text{8}},(j-1)\times \frac{\pi }{\text{4}}+\frac{3\pi }{\text{8}}\right\rfloor \end{array}\\ \frac{\text{1}}{n},\begin{array}{c} \;othrwise \end{array} \end{array}\right. \end{array}$$ (27)

Selection probabilities for the remaining directions are given by Equation (28):


$$\begin{array}{c} \delta {{\;}^{\prime }}_{ij}\left(t\right)=\frac{1-\delta_{ij}\left(t\right)}{n-1},\begin{array}{cc} & n>1 \end{array} \end{array}$$ (28)

Here, n denotes the number of *obstacle-free* directions (n>1). The *state transition probability* is modified as given by Equation (29):


$$\begin{array}{c} p_{ij}^{k}\left(t\right)=\left\{ \begin{array}{c} \frac{\tau_{ij}^{\alpha }\left(t\right)\eta_{ij}^{\beta }\left(t\right)}{\sum_{s\in allowed_{k}}\tau_{is}^{\alpha }\left(t\right)\eta_{is}^{\beta }\left(t\right)}\begin{array}{cc} \delta_{ij}^{\gamma }\left(t\right) & j\in allowed_{k} \end{array}\\ 0\begin{array}{cccc} & & & \begin{array}{cccc} & & & \;otherwise \end{array} \end{array} \end{array}\right. \end{array}$$ (29)

Among these, $\tau_{ij}\left(t\right)$ denotes the pheromone concentration along path (i,j), $\eta_{ij}\left(t\right)$ represents the enhanced potential field *force-inspired* information, and$\delta_{ij}\left(t\right)$ is a visual *interest-influenced* probability selection factor. α is a pheromone *concentration- inspired* factor: smaller values slow convergence, potentially leading to local optima, whereas larger values accelerate convergence but increase the risk of local optima. β is a potential field *force-inspired* factor: excessively small β hinders convergence speed and complicates global optimal path search, while larger β reduces randomness but also predisposes to local optima. γ signifies the visual guidance-inspired factor. With $\tau_{ij}\left(0\right)=\tau_{\text{0}}$ initialized, pheromone level updates along paths follow Equations (30),(31).


$$\begin{array}{c} \tau_{ij}(t+1)=(1-\rho )\tau_{ij}\left(t\right)+\Delta \tau_{ij}(t,t+1) \end{array}$$ (30)

$$\begin{array}{c} \Delta \tau_{ij}(t,t+1)=\sum_{k=1}^{m}\Delta \tau_{ij}^{k}(t,t+1) \end{array}$$ (31)

Among these parameters, ρ denotes the pheromone evaporation coefficient with ρ∈[0,1), and (1−ρ) represents the pheromone residue ratio. If ρ is excessively large, it may reduce the ants’ search space; conversely, if ρ is too small, it may weaken the system’s positive feedback, resulting in a more random search path. $\Delta \tau_{ij}^{k}\left(t\right)$denotes the residual pheromone deposited by ant k during the optimization process. From a broader perspective, $\tau_{ij}\left(t\right)$ illustrates how past pheromones influence the current search trajectory, while $\eta_{ij}\left(t\right)$ represents the extent to which future information affects this path. Both factors substantially affect global convergence.

This paper employs the *Max-Min Ant System* to constrain pheromone levels along paths within $\left[\tau_{min},\tau_{max}\right]$; specifically, if $\tau_{ij}<\tau_{min}$, then set $\tau_{ij}=\tau_{min}$; if $\tau_{ij}>\tau_{max}$, then set $\tau_{ij}=\tau_{max}$ instead. Additionally, the *Ant-Cycle* model is employed as defined in Equation (32).


$$\begin{array}{c} \Delta \tau_{ij}^{k}\left(t\right)=\left\{ \begin{array}{c} \frac{Q}{L_{k}},\begin{array}{cc} & \left(i,j\right)\in X_{\text{0}}^{k} \end{array}\\ 0,\begin{array}{ccc} & & \text{otherwise} \end{array} \end{array}\right. \end{array}$$ (32)

Among these variables, Q denotes the pheromone intensity, which influences convergence speed. $L_{k}$ represents the total distance traveled by ant *k* in one cycle, while $X_{\text{0}}^{k}=\{X_{\text{0}},X_{\text{1}},...,X_{k}\}$ refers to the set of nodes visited by ant *k* from the start to the endpoint during the optimization process. To enhance computational efficiency, the environmental grid can be preprocessed to obtain the pheromone diffusion grid state and construct a pheromone diffusion table. During camera operation, the corresponding grid can be queried.

The DPC algorithm is employed to facilitate the partitioning of *Traveling Salesman Problem* (TSP) instances in large-scale scenarios. By delineating the TSP area scale, different levels of path optimization can be achieved. Specifically, by interlinking local TSP instances across different groups, a hierarchical path planning problem with *high-* and l*ow-level* structures can be formulated.

The *high-level* structure refers to the connectivity between paths across distinct TSP groups, which is optimized using the *K-opt* method. The *K-opt* algorithm represents a relatively recent heuristic approach for solving the TSP. Its fundamental principle involves iteratively replacing visited nodes $X_{\text{1}},...X_{k^{\prime }}$ with unvisited nodes ${\tilde{X}}_{\text{1}},...,{\tilde{X}}_{k^{\prime }}$ under $k^{\prime }\leq k$ via a *k-change* neighborhood search, thereby producing a new path. If no further optimization can be achieved using the *K-opt* algorithm on this path, the path is considered optimal, as shown in Equation (33):


$$\begin{array}{c} \sum_{i=1}^{k^{\prime }}d_{{\tilde{X}}_{i}}<\sum_{i=1}^{k^{\prime }}d_{X_{i}} \end{array}$$ (33)

Let E represent the set of arcs forming all paths. For any arc a∈E connecting two nodes, its length is $c_{a}>0$. Let X represent the node set corresponding to all edges; E forms a directed graph G(X,E). $\sigma_{j}$ represents the candidate arcs for the replaceable path i. Consequently, the reconstructed path scheme is$S=(\sigma_{\text{0}},...,\sigma_{j},...\sigma_{K-1})$, and its cost is given by Equation (34):


$$\begin{array}{c} Z\left(S\right)=\sum_{j=0}^{K-1}d\left(h\left(\sigma_{j}\right),t\left(\sigma_{j+1}\right)\right)+\sum_{j=0}^{K-1}c_{\sigma_{j}} \end{array}$$ (34)

Here, $h\left(\sigma_{j}\right)$ and $t\left(\sigma_{j}\right)$ denote the two endpoint nodes of candidate arc $\sigma_{j}$, $d\left(h\left(\sigma_{j}\right),t\left(\sigma_{j+1}\right)\right)$ represents the shortest path length between $h\left(\sigma_{j}\right)$ and$t\left(\sigma_{j+1}\right)$ (excluding the arc), with subscripts operating modulo *K*. $\Sigma_{j=0}^{K-1}c_{\sigma_{j}}$ denotes the total edge cost along the selected path. The number of new paths Sthat can be generated is denoted as N(S), and their relationship is given by Equation (35):


$$\begin{array}{c} N(S)=(\prod_{i=1}^{K}\left|A_{R}^{i}\right|)((K-1)!) \end{array}$$ (35)

Muyldermans et al. [27] proposed a local search scheme that integrates *reverse*, *flip*, and *dir-opt* techniques, denoted as $F_{rev}(\cdot )$, $F_{flip}(\cdot )$, and $F_{dir}(\cdot )$ herein. Building upon their work, we further enhance these methods. This approach defines three fundamental search strategies: *reverse*, *flip*, and *dir-opt*.

By optimizing and integrating these basic searches, the original path is iteratively divided into new subsequences and reorganized to produce an optimized path, as defined by the following relationship: $d\left(h\left(\alpha_{i}\right),t\left(\beta_{i+1}\right)\right)$+$z\left(F_{dir}\left(F_{flip}\left(s_{i+1,r}\right)\right)\right)$+ $d\left(h\left(\beta_{i+r}\right),t\left(\alpha_{i+r+1}\right)\right)$<$d\left(h\left(\alpha_{i}\right),t\left(\alpha_{i+1}\right)\right)$ +$z\left(s_{i+1,r}\right)$+$d\left(h\left(\alpha_{i+r}\right),t\left(\alpha_{i+r+1}\right)\right)$. Forj=1,...,r, the term $z\left(s_{i+1,r}\right)$ in the aforementioned equation denotes the candidate edge necessary to maintain path continuity, as given by Equation (36):


$$\begin{array}{c} z\left(s_{i+1,r}\right)=\sum_{j=i+1}^{i+r-1}d\left(h\left(\alpha_{j}\right),t\left(\alpha_{j+1}\right)\right) \end{array}$$ (36)

This paper adopts a hybrid approach that combines the *2-opt* algorithm [27] (proposed by Levin) with the *3-opt* variant [28] (introduced by Muyldermans) to enable both path recombination within sub-scenes and path optimization across sub-scenes. The optimization process takes into account not only the path length but also various factors such as attraction to visual interests and camera rotation cost. Consequently, we adopt a *path-weighting* approach to facilitate *K-opt* optimization.

We define the configuration information of camera position points $h\left(\sigma_{j}\right)$ and $t\left(\sigma_{j}\right)$ at each arc’s two ends along the path as $\left(h\left(\sigma_{j}\right),dir\left(h\left(\sigma_{j}\right)\right)\right)$ and$\left(t\left(\sigma_{j}\right),dir\left(t\left(\sigma_{j}\right)\right)\right)$, respectively, where $dir\left(h\left(\sigma_{j}\right)\right)$and $dir\left(t\left(\sigma_{j}\right)\right)$ denote camera orientations at these positions. Subsequently, we establish a weight function for each arc, as given by Equations (37),(38).


$$\begin{array}{c} \left\{ \begin{array}{c} w_{d}\left(h\left(\sigma_{j}\right),t\left(\sigma_{j}\right)\right)=d\left(h\left(\sigma_{j}\right),t\left(\sigma_{j}\right)\right)\\ w_{r}\left(h\left(\sigma_{j}\right),t\left(\sigma_{j}\right)\right)=\frac{dir\left(h\left(\sigma_{j}\right)\right)\cdot dir\left(t\left(\sigma_{j}\right)\right)}{\left(h\left(\sigma_{j}\right)\right)∥\cdot \left(t\left(\sigma_{j}\right)\right)∥}\\ w_{v}\left(h\left(\sigma_{j}\right),t\left(\sigma_{j}\right)\right)=\underset{\begin{array}{c} h\left(\sigma_{j}\right)\in \tilde{N}\left(X_{foc},\varepsilon \right)\\ t\left(\sigma_{j}\right)\in \tilde{N}\left(X_{foc},\varepsilon \right) \end{array}}{max}\vartheta \left(X_{foc}\right)-\frac{\vartheta \left(h\left(\sigma_{j}\right)\right)+t\left(\sigma_{j}\right))}{\text{2}} \end{array}\right. \end{array}$$ (37)

$$\begin{array}{c} L_{w}\left(h\left(\sigma_{j}\right),t\left(\sigma_{j}\right)\right)=w_{d}\left(h\left(\sigma_{j}\right),t\left(\sigma_{j}\right)\right)\cdot w_{d}\left(h\left(\sigma_{j}\right),t\left(\sigma_{j}\right)\right)\cdot w_{v}\left(h\left(\sigma_{j}\right),t\left(\sigma_{j}\right)\right) \end{array}$$ (38)

Among these variables, $w_{d}\left(h\left(\sigma_{j}\right),t\left(\sigma_{j}\right)\right)$,$w_{r}\left(h\left(\sigma_{j}\right),t\left(\sigma_{j}\right)\right)$, and$w_{v}\left(h\left(\sigma_{j}\right),t\left(\sigma_{j}\right)\right)$ denote weights for arc distance (between $h\left(\sigma_{j}\right)$ and $t\left(\sigma_{j}\right)$), camera rotation, and viewpoint quality, respectively. $X_{foc}$ denotes the focal point of the visual interest group including $h\left(\sigma_{j}\right)$ and $t\left(\sigma_{j}\right)$, specifically the region containing the building with optimal viewpoint quality.

The arc connecting $h\left(\sigma_{j}\right)$ and $t\left(\sigma_{j}\right)$ is denoted as a. Consequently, our optimization objective is to minimize $L_{w}\left(a\right)$, as given by Equation (39):


$$\begin{array}{c} l_{min}\left(a\right)=\underset{h\left(\sigma_{j}\right),t\left(\sigma_{j}\right)\in a}{argmin}L_{w}\left(a\right) \end{array}$$ (39)

Building on the hierarchical TSP algorithm [29], which employs density peak clustering and ant colony optimization, this paper reformulates the path planning problem—initially based on potential field force and visual regions of interest—into a *K-opt* path optimization task. By integrating the *3-opt* algorithm as an enhancement, we obtain an optimized path. Unlike traditional path planning, our method prioritizes capturing comprehensive global visual information rather than conventional metrics such as shortest distance or convergence time. Exploring virtual environments is complex, as the optimal path should prioritize visually rich content over minimal length. While maximizing visual information is crucial, the path must also be as short as possible, which is a significant challenge.

## 3. Experimental design and analysis

In path optimization, several robust and implementable algorithms are widely employed, including *Genetic Algorithm* (GA), *Particle Swarm Optimization* (PSO), *Grey Wolf Optimization* (GWO), *Ant Colony Optimization* (ACO), and *Beetle Antennae Search* (BAS).

Previous studies have shown that GA and GWO demonstrate strong convergence and computational simplicity, whereas ACO and PSO are characterized by fast search speeds, high robustness, and low dependence on initial conditions. Overall, these algorithms are favored for their global search capability, convergence performance, algorithmic simplicity, and stability under varying environmental conditions.

### 3.1. Experimental methods and parameter design

To establish a rigorous experimental framework, we first conducted a systematic literature review to synthesize diverse methodological advancements. Subsequently, this study proposes an integration of a *field-oriented* ant colony algorithm with viewpoint guidance. To assess its effectiveness, comparative experiments were conducted using four widely adopted optimization algorithms: *Ant Colony System* (ACS), *Genetic Algorithm* (GA), *Grey Wolf Optimizer* (GWO), and *Beetle Antennae Search* (BAS).

#### 3.1.1. Rationale and sourcing for Ant Colony System (ACS) parameter settings.

**Core parameter roles in ACS:** Path selection within the ACS algorithm is governed by three interdependent parameters, whose nonlinear interactions directly determine algorithmic exploration capability, convergence performance, and operational stability (see Table 1):

- Pheromone factor (*α*): Modulates the influence of pheromone accumulation at discrete nodes; higher values increase the algorithm’s reliance on historical path information to enhance exploitation.
- Heuristic weight (*β*): Directs the algorithm toward heuristically favorable paths (e.g., shorter distances), balancing the need to avoid excessive exploitation or random exploration.
- Evaporation coefficient (*ρ*): Governs the pheromone decay rate, acting as a critical trade-off parameter—higher values promote exploration (by reducing the influence of outdated pheromones), whereas lower values accelerate convergence (by reinforcing successful paths).

**Table 1:** Parameter settings of the ACS method compared with other methods.

Parameters	Test Group 1	Test Group 2	Test Group 3	Test Group 4	Test Group 5	Reference Value
*α*	0	1	2	3	4	1
*β*	0	1	3	4	5	3
*ρ*	0.1	0.15	0.25	0.4	0.5	0.25

**Literature-driven initial parameter sourcing:** To ensure consistency with domain benchmarks, the initial ACS parameter values were drawn from three categories of influential studies:

(1) Foundational ACS parameter studies

Preliminary ranges for *α*, *β*, and *ρ* were established based on studies investigating parameter sensitivity in path planning scenarios analogous to the present work:

- Wang et al. [30]: Tested *α*/ *β*/ *ρ* combinations for 3D ground robot path planning, providing baseline ranges for *obstacle-cluttered* environments.
- Ma et al. [31]: Explored ACS parameter tuning for underwater navigation, emphasizing *ρ*’s role in balancing convergence speed and path optimality.
- Sangeetha et al. [32,33]: Proposed reference values for *α*, *β*, and *ρ* in dynamic ACO using *fuzzy logic* and *intelligent gain* methods, offering theoretical support for initial parameterization.

(2) Focused parameter-tuning studies

Refinements to initial values were guided by studies explicitly optimizing ACS parameters for complex environments:

- Yue et al. [34]: Simulated multi-obstacle scenarios and validated *α* = 1, *Q*= 100 (pheromone intensity), *β* = 4, *γ* = 2 (motivational factor), and *ρ* = 0.25 as a high-performance combination.
- Perez-Carabaza et al. [35]: Detailed ACS implementation protocols for comparative experiments, confirming *α* ∈ [1,2] and *ρ* ∈ [0.2, 0.3] as robust defaults for 3D path planning.
- Sangeetha et al. [36,37]: Their “*green ant colony*” and *intelligent gain* approaches further supported *β* ∈ [3,4] to prioritize heuristic path guidance without sacrificing exploration.

(3) Cross-disciplinary validation from related ACO variants

Further insights were drawn from recent ACO-based path planning studies to ensure generalizability:

- Liu et al. [38]: Applied potential field-enhanced ACO to population evacuation path optimization, validating *γ* = 2 as an effective *motivational factor*.
- Liang et al. [39]: Used context-aware ACO for tourism route planning, confirming *α* ∈ [1,2] and *β* ∈ [3,4] as balanced for multi-objective optimization.
- Zhang et al. [40]: Optimized 3D continuous picking paths with ACO, reinforcing *ρ* = 0.25 as optimal for reducing computational overhead while maintaining path quality.

**Large-scale scene and evaporation factor (*ρ*) validation:** To confirm parameter suitability for large-scale digital twin environments, we reviewed studies addressing scalability and *ρ*-specific performance:

- Caprio et al. [41]: Analyzed ACO convergence in *fuzzy optimal path planning*, confirming *ρ* ∈ [0.2, 0.3] minimizes convergence time in large environments.
- Yang et al. [42]: Proposed a game theory-based ACO for the *Traveling Salesman Problem* (TSP), validating *α* = 1, *β* = 3, and *ρ* = 0.25 as a stable combination for high-dimensional spaces.
- Zhang et al. [43,44]: Used Taguchi’s method (pre-experiments, orthogonal testing, and simulation validation) to optimize dynamic multi-role ACO, confirming *τ*₀ = 0.05 (initial pheromone) as optimal for preventing premature convergence.
- Ajeil et al. [45]: Specifically evaluated *ρ* and found that *ρ* ∈ [0.3, 0.7] degrades computational efficiency; their 10-replicate simulations confirmed *ρ* = 0.25 balances performance and speed.

#### 3.1.2. Parameter sourcing for Grey Wolf Optimizer (GWO).

GWO parameters were iteratively refined through two sequential phases of literature review:

(1) **Initial phase**

Rao et al. [46] (*mobile robot navigation tasks*) provided baseline population size and convergence coefficients; Jabbarpour and Fu [47] (*comparative GWO experiments*) validated these values; Yu et al. [48] (*hybrid multi-objective path planning research*) supplemented coefficient tuning rules.

(2) **Refinement phase**

Recent advances in GWO optimization further informed settings:

- Bai et al. [49]: Improved GWO for feature selection, confirming a population size of 100 as optimal for complex 3D environments.
- Zhang et al. [50]: Adaptive self-learning GWO validated the coefficient update rule: *a* = 2×(1 − *t*/*T*) (where *t* denotes the current iteration and *T* denotes the maximum number of iterations).
- Chen et al. [51] (golden-section GWO) and Liu et al. [52] (*improved GWO for mobile robots systems*) confirmed random coefficient ranges: *A* = Rand(-*a*, *a*) and *C* = Rand(0, 2), with probability *P* = exp(-∆*f*/*T*) (∆*f* denotes the fitness difference) for exploration control.

#### 3.1.3. Parameter sourcing for Genetic Algorithm (GA).

The parameters of the Genetic Algorithm (GA) were defined based on widely accepted domain standards and further refined with reference to recent state-of-the-art studies. Specifically, the algorithm employs one-point crossover as the crossover design, with a population size of 200 genomes, a crossover probability of 70%, a mutation probability of 30%, and a termination condition set at 120 iterations.

(1) **Initial settings**

Zhang et al. [50] (*multi-objective path planning*) and Zhou et al. [53] (*standard GA parameter benchmarks*) established a crossover probability (*P*_*c*_) = 0.7 and a mutation probability (*P*_*m*_) = 0.3.

(2) **Refinement**

Liu et al. [54] (*machine learning-based GA parameterization*) and Nagasawa et al. [55] (*GA-neural network comparisons*) confirmed that *P*_*c*_ = 0.7 and *P*_*m*_ = 0.3 prevent premature convergence.

(3) **Validation**
  - Aivaliotis-Apostolopoulos et al. [56]: ACS-GA comparisons validated a population size of 200 chromosomes for 3D path planning.
  - Alharthi et al. [57]: Deep reinforcement learning-integrated GA reinforced *single-point crossover* as optimal for path encoding.
  - Qi et al. [58] (*UAV path planning*) and An et al. [59] (*3D obstacle avoidance benchmarks* involving GA, PSO, AC, and GWO) confirmed all GA settings as robust for complex environments.

#### 3.1.4. Parameter sourcing for Beetle Antennae Search (BAS).

BAS, a metaheuristic inspired by beetle foraging behavior, was parameterized based on the following sources:

(1) **Foundational values**

Jiang et al. [60] (*ACO-BAS* comparative experiments) provided core parameters, including an initial step size (*δ*) = 8, an evaluation step size (*θ*) = 21, a speed constant (*λ*1) = 0.998, an experience constant (*λ*2) = 0.998, and a change constant (*c*) = 2.

(2) **Convergence validation**

Recent advances in BAS research confirmed these parameter settings:

- Chen et al. [61]: Conducted a convergence analysis of BAS in optimization tasks and validated *δ* = 8 and *θ* = 21 for high-dimensional 3D search spaces.
- Zhao et al. [62] (*inertia-weighted BAS*), Shan et al. [60] (*hybrid BAS framework*), Zhang et al. [63] (*BAS convergence mechanism research*), and Ye et al. [64] (*multi-operator improved BAS*) all reinforced *λ*1 = *λ*2 = 0.998 and *c* = 2 as effective choices for balancing exploration and exploitation capabilities.

#### 3.1.5. Final parameterization for all algorithms and fair comparison protocols.

(1) **Optimized parameters** **for the proposed method**

The vision-guided potential field ant colony algorithm’s final parameters were determined via literature validation and iterative simulation (testing *α* ∈ {0, 1, 2, 3, 4}, *β* ∈ {0, 1, 3, 4, 5}, *γ* ∈ {0.5, 1.5, 2, 3, 5}, *ρ* ∈ {0.1, 0.15, 0.25, 0.4, 0.5}, *τ*₀ ∈ {0.01, 0.03, 0.05, 0.08, 0.15}):

- Number of ants: 200 (a trade-off between global search and computational efficiency, as discussed in Section 3.1.3).
- *α* = 2, *β* = 3, *γ* = 2, *ρ* = 0.25, *τ*₀ = 0.05 (identified as optimal for minimizing direction changes and overlapping trajectories in 3D digital twin environments).

(2) **Control algorithm parameters**

To ensure a fair comparison, all control algorithms were parameterized to match domain benchmarks (as summarized in Table 2):

**Table 2:** Comparison of parameter settings for different methods.

Algorithm	Key Parameters	Rationale (Literature Source)
ACS	Number of ants = 200; *α* = 1, *β* = 3, *ρ* = 0.25; *τ*_0_ = 1; Local evaporation rate (*ξ*)=0.3	Kang et al. [38]; Yue et al. [30]
GA	Population size = 200; Single-point crossover; *P*_*c*_ = 0.7, *P*_*m*_ = 0.3	Zhang et al. [49]; An et al. [55]
GWO	Population size = 100; *a* = 2×(1 − t/T); *A* = Rand(-*a*,*a*), *C* = Rand(0,2); *P* = exp(-∆*f*/*T*)	Bai et al. [45]; Zhang et al. [46]
BAS	Population size = 60; *δ* = 8, *θ* = 21; *λ*1 = 0.998, *λ*2 = 0.998, *c* = 2	Jiang et al. [56]; Chen et al. [57]
Proposed Method	Number of ants = 200; *α* = 2, *β* = 3, *γ* = 2, *ρ* = 0.25, *τ*_0_ = 0.05$\eta_{ij}\left(t\right)=a^{F_{tot}cos\theta_{F}}/d^{\text{2}}\left(X_{i},X_{j}\right)$	Zhang et al. [36]; Zhang et al. [40]; Ajeil et al. [41];

#### 3.1.6. Experimental protocols for fairness.

All algorithms (the proposed method and four control methods) were tested under identical conditions:

- *Maximum iterations*: 120 (consistent with large-scale 3D path planning studies [40,44]).
- *Simulation environment*: An identical 3D virtual digital twin environment (to eliminate environmental bias).
- *Replicates*: 10 independent simulations per algorithm (to ensure result reliability, per Ajeil et al. [45]).

### 3.2. Experimental results

**Fig 1:**
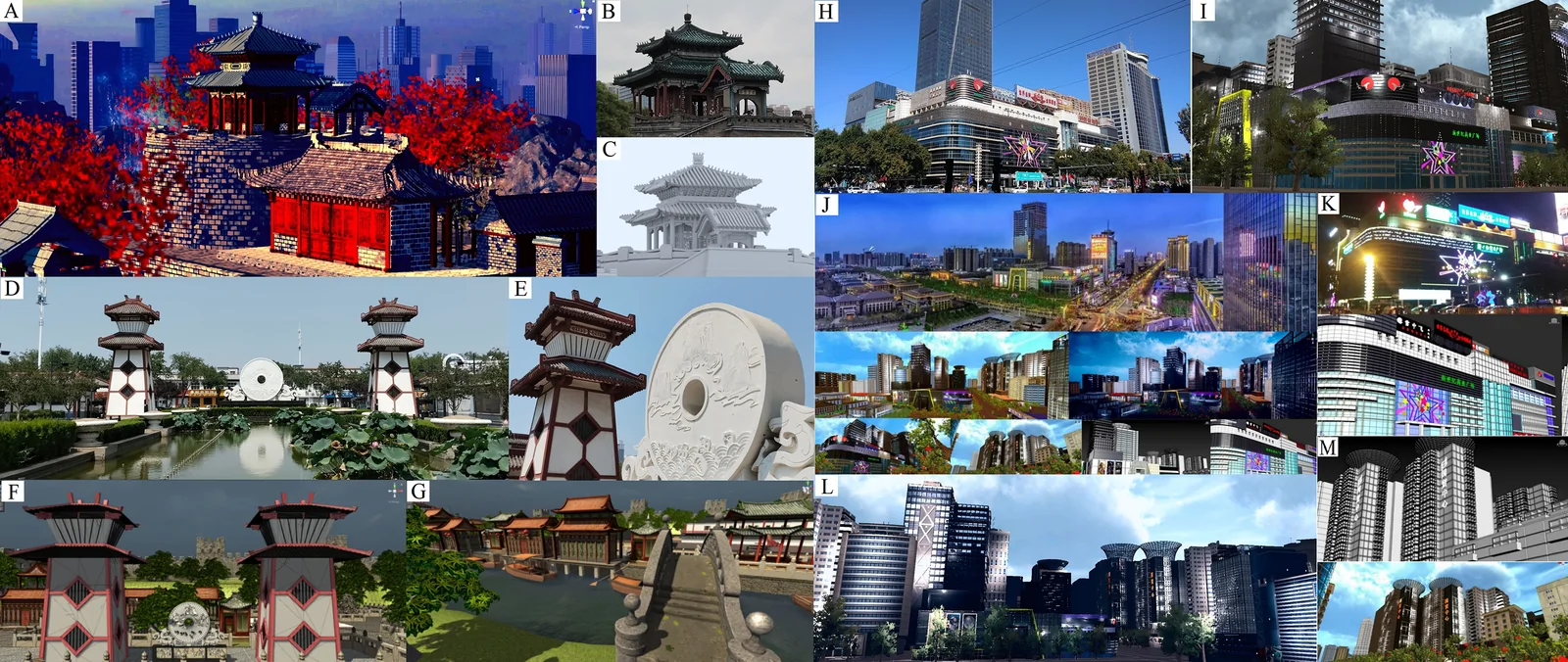
Overview of constructed digital twin environments: (A)-(C) small-scale heritage simulation based on Congtai park, (D)-(G) large-scale digital twin scene derived from Zhaoyuan heritage park, and (H)-(M) complex urban virtual environment reconstructed from Handan New Century Plaza.

This study focuses on the aesthetics of virtual garden landscapes, using a digital twin scene constructed from classical garden landscape data. Future research will extend the proposed framework to broader application scenarios, such as digital cities. In the first experiment (*Scene 1*), we employed a complex scene inspired by Chinese classical garden aesthetics—the *Congtai* ruins park (as illustrated in Fig 1(A)–(C)). The site features scattered, small-scale buildings with intricate details (e.g., wooden *dougong*, upturned eaves). Its layout embodies key characteristics of northern Chinese classical gardens: remoteness, winding pathways, and aesthetic appeal, while maintaining a moderate scale and complexity. This setting is ideal for evaluating how different exploration methods affect scene understanding, particularly the link between visual perception and aesthetic experience during navigation.

**Fig 2:**
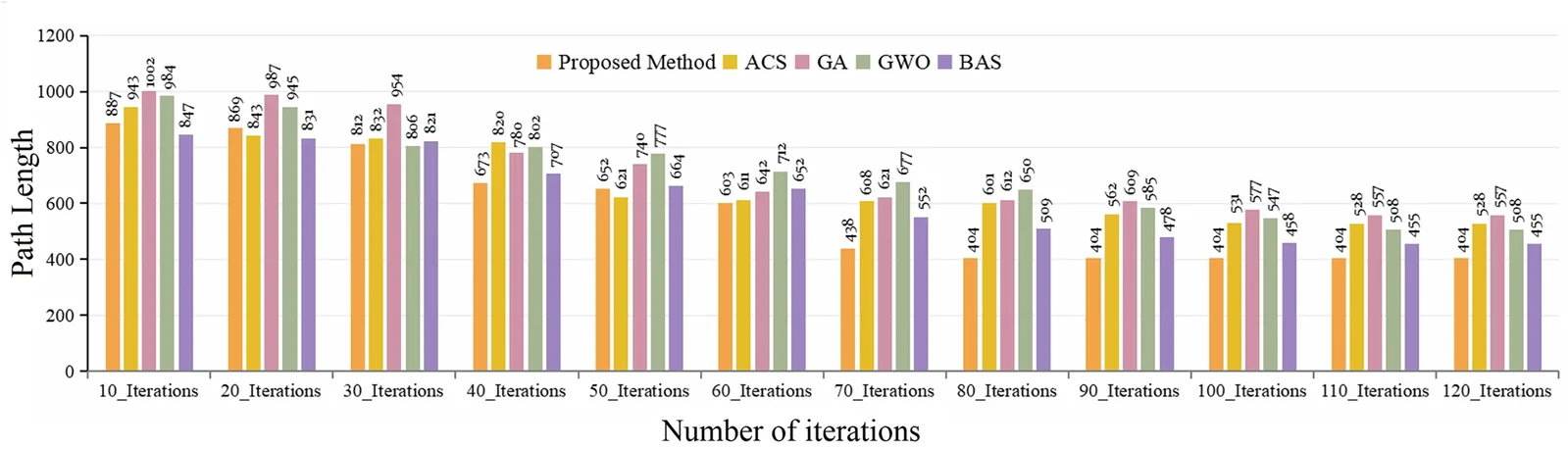
Path length after iterative convergence (*Scene 1*).

For *Scene 1*, we constructed a digital twin environment of *Congtai* park (see Fig 1). The simulated *Congtai* ruins environment comprises 18 buildings and 16 garden landscape nodes. In *Scene 1* (*Congtai* Park Digital Twin), we conducted a comparative experiment using the proposed methodology (hereafter referred to as the proposed method). The results show that the proposed method converges to the optimal path (length = 404.37) after more than 80 iterations. The iteration information for ACS, GA, and GWO is shown in Fig 2. As the number of iterations increases, the proposed method outperforms the other comparative methods. In path optimization, it achieves improvements of 23.48% (ACS), 27.47% (GA), 20.47% (GWO), and 11.21% (BAS), as shown in Fig 2.

**Fig 3:**
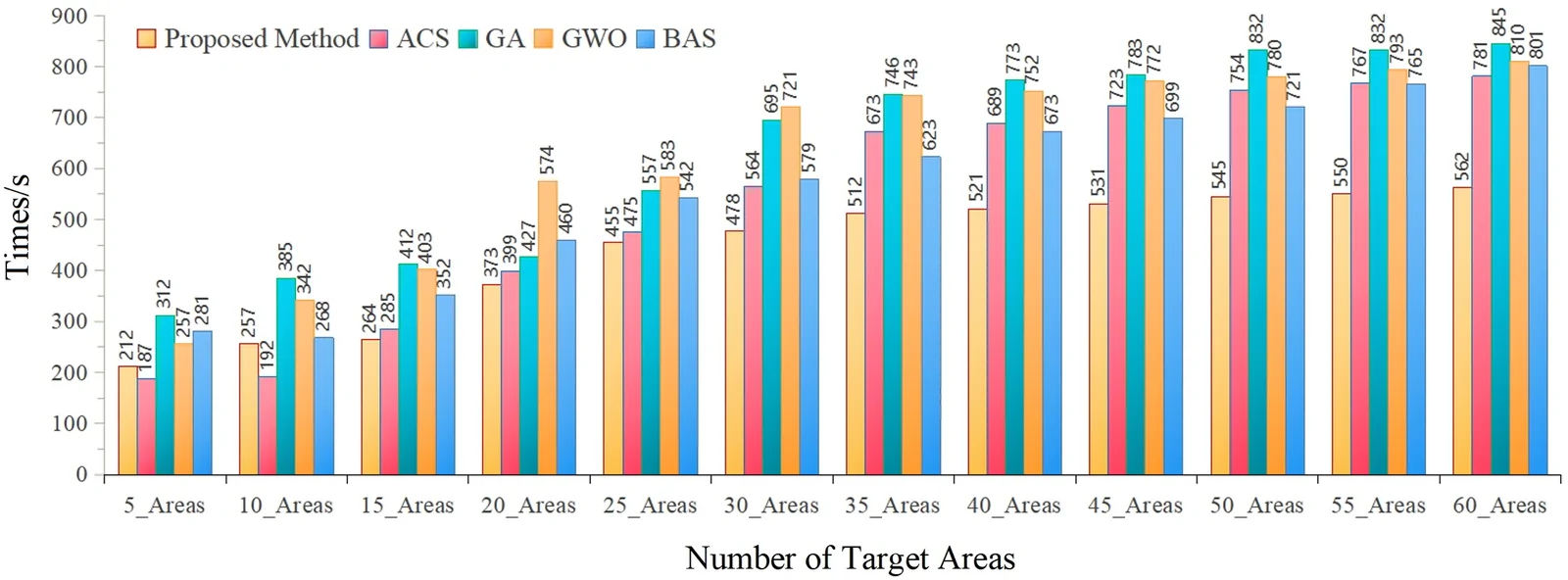
Exploration time consumption for the target area in *Scene 1.*

We executed 120 iterations for different search strategies, and the results are shown in Fig 3. Compared to the *genetic algorithm* (GA)’s final solution, the proposed method converged around iteration 80, while GA required approximately 100 iterations to converge. Our approach’s convergence speed significantly outperforms the other four examined methods.

For ACO, its performance is primarily influenced by the control parameters *α*, *β*, and *ρ*; therefore, multiple parameter combinations were established to evaluate ACO’s sensitivity to multi-objective function values and iteration counts. The exploration of different methods in the target area of the virtual environment is shown in Fig 3.

For *Scene 1*, the proposed method achieves an optimal path length of 404.37. The optimal path lengths of the baseline algorithms are listed as follows: ACS = 528.47, GA = 557.27, GWO = 508.42, and BAS = 455.16. The simulation results of the five methods for exploring the target area in *Scene 1* are presented in Fig 3. The results reveal that by the end of the simulation, the proposed *visual-guided* potential field ant colony algorithm requires significantly less time to explore 55 scene sub-regions than ACS, GA, GWO, and BAS (see Figs 3). Among the four comparative methods, GA demonstrates the highest area exploration time efficiency, ranking second only to the proposed method.

**Fig 4:**
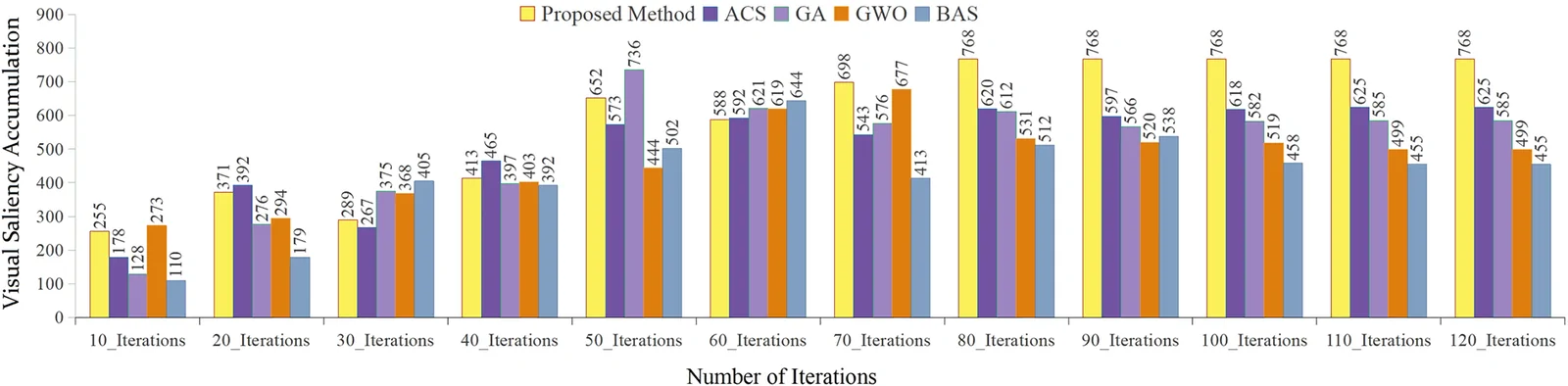
Accumulated visual saliency after iterative convergence in *Scene 1.*

The experimental results confirm that the proposed method achieves high efficiency in exploring specific target areas. In contrast, ACS consistently exhibits low search efficiency. Although its performance is comparable to that of other methods, none of them achieves particularly strong performance. By the end of the simulation—particularly in tasks involving visually rich scene objects—the proposed method outperforms ACS, GA, GWO, and BAS in both speed and efficiency, with significantly shorter exploration times. As shown in Fig 4, in smaller scenes (with 60 target objects), the proposed method reduces exploration time by 28.04% over ACS, 33.49% over GA, 30.62% over GWO, and 29.84% over BAS. These results demonstrate that the proposed method explores target areas more rapidly than existing approaches, thereby significantly enhancing overall search efficiency.

Visual saliency accumulation during scene roaming is a core metric for evaluating algorithm effectiveness. After convergence, the visual saliency collected by the virtual camera is presented in Fig 4. The proposed method converges at approximately 80 iterations — earlier than the four baseline methods — and achieves the highest accumulated visual saliency. It outperforms ACS, GA, GWO, and BAS in visual saliency accumulation, with improvements of 22.89%, 31.29%, 53.91%, and 68.79%, respectively, after 120 iterations. The comparative methods often miss *visually salient areas* (see Fig 4), leading to suboptimal paths that fail to maximize *saliency*. Among them, ACS performs best, while BAS performs the worst. In *Scene 2*, we constructed a digital twin of *Zhaoyuan* ruins park, including 37 large-scale buildings and 35 garden landscape nodes, which match the park’s original layout and digital twin standards. It features diverse classical garden structures (e.g., pavilions, terraces, and towers) that enhance visual richness and aesthetic quality, as shown in Fig 1(D)–(G).

Users may feel disoriented when freely navigating such an expansive scene, and individuals lacking in-depth knowledge of classical gardens may find it challenging to comprehend the structural hierarchy within this environment. Consequently, this environment serves as an ideal virtual reality setting for preliminary experiments. To enhance the virtual environment’s complexity and scale, we increased the number of buildings from 37 to 55 and the number of garden landscape nodes to 51, resulting in over 120 exploratory areas in this enhanced environment. Additionally, we conducted comparative experiments using various methods (ACS, GA, GWO, and BAS) within this scene, mitigating potential biases arising from a singular visual style of scene resources. The selected scene not only has significant volume and complexity but also features a visual style markedly different from the classical garden-style virtual environment used in *Scene 1*. Thus, this intricate setting serves as an effective test environment for assessing various indicators across different visual styles and levels of scene complexity.

For the formal experiment, we distributed the experimental questionnaire via the *Sojump* platform, whose data are considered reliable for research. Due to the scene’s extensive scale, intricate building cluster organization, and relatively complex structural hierarchy, we uniformly increased the iteration number of the five comparative methods.

**Fig 5:**
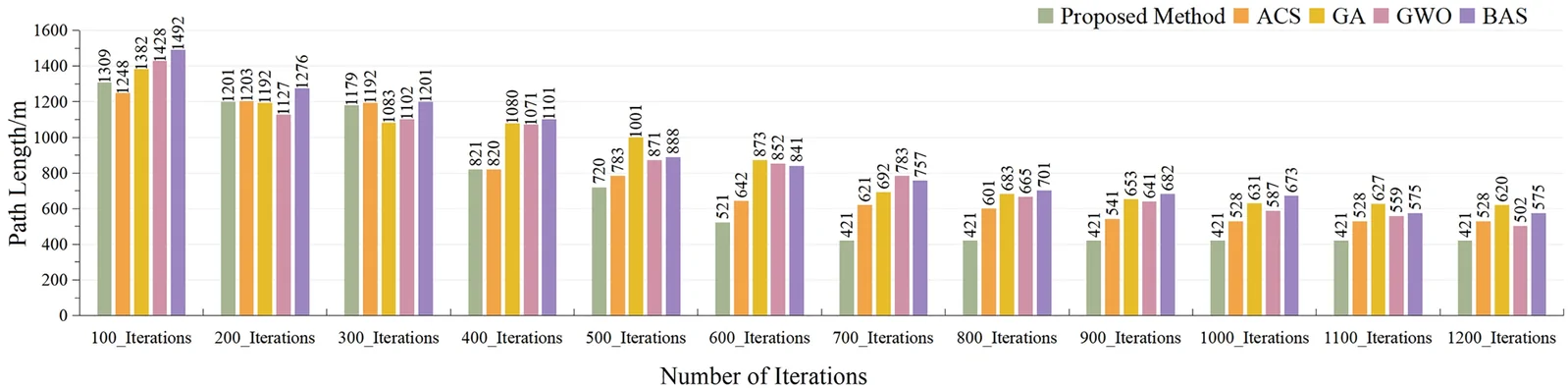
Path length after iterative convergence in *Scene 2.*

Experimental results reveal that the proposed method converges after approximately 700 iterations, achieving the shortest optimized path. GWO is effective but requires more iterations to converge. In contrast, GA produces the longest path among the five comparative algorithms and demonstrates the least effective path optimization (as shown in Fig 5).

**Fig 6:**
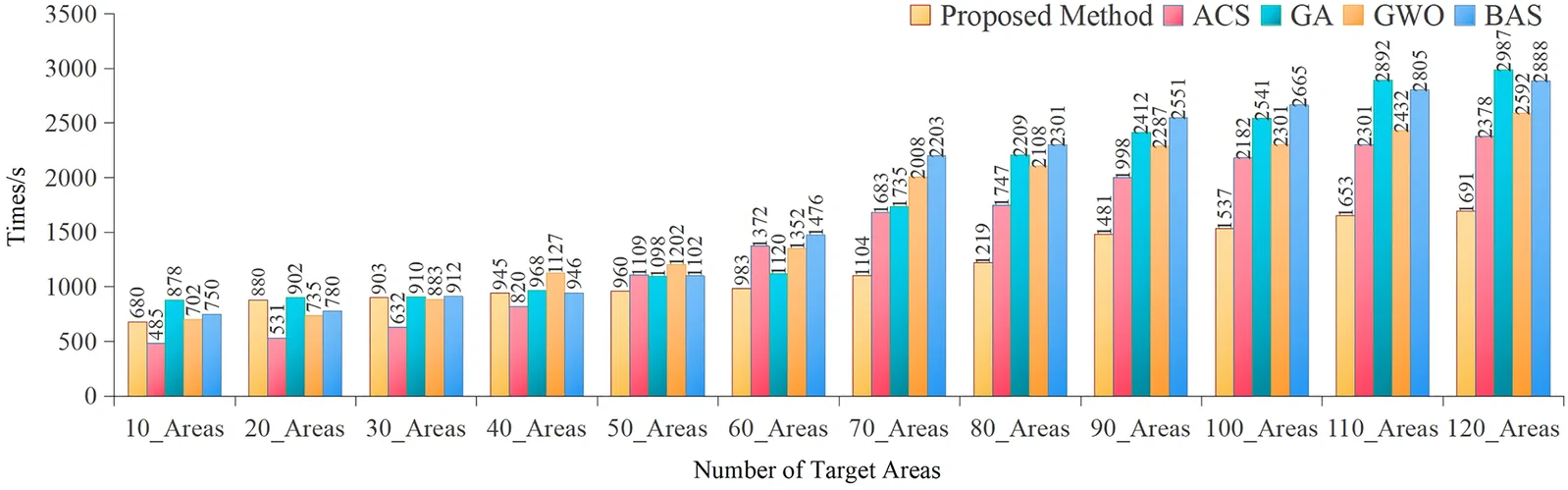
Exploration time consumption for the target area in *Scene 2.*

In *Scene 2*, comparative experiments between our method and the four optimization algorithms (ACS, GA, GWO, and BAS) indicate that, under the same scene scale and complexity, GWO requires the longest convergence time, followed by BAS. GA and ACS exhibit similar convergence times, while the proposed method achieves the fastest convergence (see Fig 5). For the optimal path length, BAS produces the longest path, while GWO and GA yield intermediate-length paths. The proposed method obtains the shortest path with fewer iterative iterations, outperforming ACS (path length reduced by 20.27%), GA (reduced by 32.09%), GWO (reduced by 16.14%), and BAS (reduced by 26.78%) in path length in *Scene 2*. In terms of target object exploration efficiency, our method has a clear advantage. As shown in Fig 6, in the larger and more complex *Scene 2* (with over 120 target areas), our method reduces exploration time by 28.89% (ACS), 43.39% (GA), 34.76% (GWO), and 41.45% (BAS), while converging faster and achieving the shortest optimized path.

**Fig 7:**
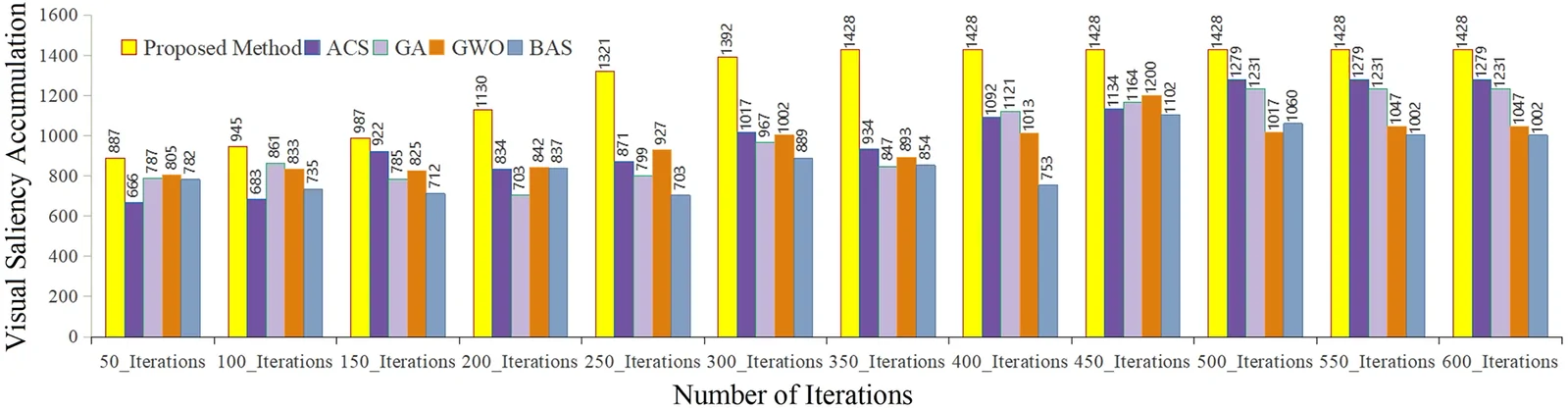
Accumulated visual saliency after iterative convergence in *Scene 2.*

During iterative convergence, the proposed method exhibits a pronounced advantage in visual saliency accumulation, substantially outperforming the four comparative methods. Among these, ACS and GA achieve comparable and moderate visual saliency accumulation, whereas GWO and BAS exhibit relatively lower accumulation. Upon convergence, the proposed method increases visual saliency accumulation by 11.65% relative to ACS, 16.03% relative to GA, 36.39% relative to GWO, and 42.51% relative to BAS, as shown in Fig 7.

To assess aesthetic perception in the optimized path scenes, we employed a questionnaire adapted from landscape roaming aesthetic assessment instruments designed for *Scene 1* and *Scene 2*. The experimental procedure was designed as follows: Camera viewport data from different path planning methods were rendered into landscape animation videos. These videos were shared with participants via the *Sojump* platform to assess how various path planning approaches affect scene understanding and aesthetic ratings. Responses were collected using a *7-point semantic differential scale*.

The questionnaire comprises four sections. First, participants read instructions outlining the study’s purpose, duration, and guidelines. Second, they viewed roaming videos generated by five methods (including a *visual-perception-guided* intelligent path planning method) for *Scene 1* and *Scene 2*, with interactive questions during playback to ensure engagement. Third, the core section assessed five constructs—*Smoothness Level*, *Scene Understanding*, *Visual Richness*, *Aesthetic Intention*, and *Degree of Visual Interest*—using scales adapted from established instruments, with precautions taken to avoid bias from manipulation checks. Fourth, demographic information (gender, age, education level, and familiarity with VR technology) was collected. To ensure response quality, IP detection, behavioral verification, and location authorization were implemented prior to survey release. The questionnaire was anonymous, and participants received compensation (custom souvenirs and virtual cultural tourism badges) upon completion to incentivize participation. All measured variables were based on validated scales modified for the context of this study. Detailed definitions and item descriptions of all measurement scales are presented below.

### 3.3. Scale design and analysis

Given that this study primarily investigates how visual perception factors in complex scenes influence scene navigation, incorporating aesthetic evaluation measures is essential. To achieve this, we employ a questionnaire-based scaling approach, drawing on established methodologies from prior research, to enable a comparative analysis of different intelligent path planning methods, particularly with respect to aesthetic factor scores. The subsequent section elaborates on measurement scale design and illustrates the corresponding experimental procedures in detail.

#### 3.3.1. Scale design.

To quantitatively evaluate user experience with the proposed roaming scheme, a questionnaire was developed covering five core perceptual dimensions derived from existing literature and practical domain requirements: Smoothness Level, Scene Understanding, Visual Richness, Aesthetic Intention, and Visual Interest. Each dimension was operationalized using multiple measurement items to capture subtle perceptual nuances throughout scene exploration. Detailed scale items and corresponding coding labels are presented in Table 3.

**Table 3:** Measurement items of the five perceptual constructs.

Variable	Encoding	Measurement Items
*Smoothness Level*	SL1	The roaming path formed by this method will not have severe turning or camera shaking.
	SL2	The path is smooth and does not overlap.
	SL3	The landscape sequence formed by the roaming path is natural and smooth.
*Scene Understanding*	SU1	This method helps users understand the architectural forms in the scene.
	SU2	This method helps users better understand the overall scene layout.
	SU3	This method enables users to clearly understand the hierarchical structure of the scene.
	SU4	This method leaves users with a strong impression of landmark buildings.
	SU5	This method enables users to form a clear memory of landscape nodes in the scene.
*Visual Richness*	VR1	This method presents a visually rich building layout in the scene.
	VR2	This method presents a visually rich configuration of plants in the scene.
	VR3	This method presents rich visual features in water elements and hard landscape forms.
	VR4	This method presents a visually rich composition of landscape colors.
*Aesthetic Intention*	AI1	This method can enhance the immersive experience of scene roaming.
	AI2	This method can enhance visual comfort during scene roaming.
	AI3	This method can generate more aesthetically pleasing landscape sequences.
	AI4	This method can enhance users’ understanding of the aesthetic features of the scene.
*Visual Interest Degree*	VD1	This method arouses users’ interest in the spatial organization of buildings.
	VD2	This method creates a desire in users to explore landscape elements.
	VD3	This method enables users to capture visual information of the scene. more comprehensively.
	VD4	This method stimulates users’ willingness to explore the corresponding real scenic areas.
	VD5	This method encourages users to have a positive evaluation of the visual effects of scene roaming.

(1) Measurement of the *Smoothness Level Scale*

To quantify the aesthetic ratings of viewpoints in scene roaming videos generated by different path optimization strategies, we designed a *Smoothness Level scale* drawing on the research of Fridin and Belokopytov [65]. The scale was adapted to the context of the present study. A three-item scale was developed and rated on a *7-point Likert scale* (1 = strongly disagree, 7 = strongly agree).

(2) Measurement of the *Scene Understanding Scale*

Five measurement items were developed in this scale, and a *7-point Likert scale* was adopted for subjective evaluation (1 = not at all, 7 = extremely).

(3) Measurement of the *Visual Richness Scale*

This scale was designed to investigate participants’ perceptions of the visual richness of scene elements under different path planning conditions. We developed a four-item *Visual Richness scale* using a *7-point Likert scale* (1 = extremely poor, 7 = extremely good), consistent with the measurement of other variables.

(4) Measurement of the *Aesthetic Intention Scale*

To evaluate users’ aesthetic intention and preference toward scenes derived from different path planning schemes, we constructed a four-item scale measured on a *7-point Likert scale* (1 = not at all, 7 = very much).

(5) Measurement of the *Visual Interest Degree Scale*

The development of the *Visual Interest scale* referred to prior user experience scale studies by Gray, Wegner et al. [66]. A 5-item scale was developed, measured on a *7-point Likert scale* (1 = not at all, 7 = very much).

#### 3.3.2. Reliability and validity analysis.

In this study, *Cronbach’s alpha* coefficient was used to assess the reliability of each scale, with analyses conducted using SPSS software. Following Nunnally’s criteria, a *Cronbach’s alpha* greater than 0.9 indicates excellent reliability, values between 0.7 and 0.9 indicate good reliability, values between 0.35 and 0.7 indicate acceptable reliability, and values below 0.35 indicate insufficient reliability. The *Cronbach’s alpha* coefficients for the *Smoothness Level*, *Scene Understanding*, *Visual Richness*, *Aesthetic Intention*, and *Visual Interest Degree* scales were 0.857, 0.793, 0.887, 0.831, and 0.843, respectively, all exceeding the commonly accepted threshold of 0.7. These results demonstrate strong internal consistency of the measurement scales, supporting their reliability for subsequent statistical analyses (see Table 4).

**Table 4:** Reliability analysis of overall variables in the questionnaire sample.

Scale Items	Coding	CITC	Cronbach’s α
Smoothness Level	SL1	0.607	0.857
	SL2	0.553	
	SL3	0.546	
Scene Understanding	SU1	0.632	0.793
	SU2	0.563	
	SU3	0.551	
	SU4	0.601	
	SU5	0.537	
Visual Richness	VR1	0.547	0.887
	VR2	0.623	
	VR3	0.605	
	VR4	0.615	
Aesthetic Intention	AI1	0.623	0.831
	AI2	0.682	
	AI3	0.714	
	AI4	0.673	
Visual Interest Degree	VD1	0.589	0.843
	VD2	0.631	
	VD3	0.667	
	VD4	0.581	
	VD5	0.563	

Reliability refers to the consistency and stability of measurements. All scale items exhibited *corrected item-total correlations* (CITC) greater than 0.4, satisfying commonly accepted reliability criteria. *Cronbach’s α* ranged from 0.7 to 0.9 across dimensions, indicating strong internal consistency and confirming the reliability of the measurement instrument (see Table 10). Validity reflects the extent to which a measurement captures its intended construct [67,68]. To assess construct validity, *exploratory factor analysis* (EFA) was conducted using SPSS 27.0.1. The *Kaiser-Meyer-Olkin* (KMO) measure and *Bartlett’s test* of sphericity were employed to evaluate the suitability of the data for factor analysis: a KMO value greater than 0.7 and a significant *Bartlett’s test* (*p* < 0.05) indicate adequacy. In the present study, the KMO value was 0.884 (well above the threshold) and *Bartlett’s test* was highly significant (*p* < 0.01), confirming that the data met EFA assumptions and supporting the structural validity of the scales (see Table 5).

**Table 5:** KMO measure of sampling adequacy and *Bartlett’s Test of Sphericity.*

KMO Measure of Sampling Adequacy		0.884
	Approx. Chi-Square	1306.218
***Bartlett’s Test of Sphericity***	df	274
	Sig.	<0.01

Convergent and discriminant validity were further evaluated to assess the psychometric properties of the measurement model [69–73]. Composite reliability (CR) values greater than 0.700 indicated satisfactory internal consistency, supporting the reliability of latent constructs. These results verify adequate unidimensionality and item-level coherence of the scale, demonstrating the robustness of the overall measurement framework (see Table 6).

**Table 6:** Validity assessment of overall constructs in the questionnaire sample (Note: the standardized factor loading is denoted by *λ;* Variable coding: SL – *Smoothness Level*, SU – *Scene Understanding*, VR – *Visual Richness*, AI – *Aesthetic Intention*, VD – *Visual Interest Degree*).

Metric	SL	SU	VR	AI	VD
**Items Number**	3	5	4	4	5
***λ***_**1**_	0.864	0.848	0.832	0.887	0.768
***λ***_**2**_	0.827	0.838	0.798	0.838	0.821
***λ***_**1**_	0.816	0.812	0.784	0.807	0.779
***λ***_**3**_	―	0.826	0.825	0.868	0.712
***λ***_**4**_	―	0.809	―	―	0.887
**AVE**	0.699	0.681	0.656	0.723	0.633
**CR**	0.874	0.915	0.884	0.913	0.896

#### 3.3.3. Correlation analysis.

This study aims to investigate how various factors influence visual interest. Spearman’s rank-order correlation analysis was employed to examine significant associations among variables and to elucidate their underlying relationships. All analyses were conducted using SPSS 27.0.1 to systematically assess interrelationships among multidimensional variables. The results are summarized in Table 7.

**Table 7:** Correlation analysis (Note: Correlations are statistically significant at the 0.01 level (p < 0.01, *two-tailed Pearson correlation analysis*). Variable coding: 1 – *Smoothness Level*, 2 – *Scene Understanding*, 3 – *Visual Richness*, 4 – *Aesthetic Intention*, 5 – *Visual Interest Degree*).

Variable	1	2	3	4	5
**1**	1				
**2**	.189**	1			
**3**	.171**	.591**	1		
**4**	.573**	.238**	.164**	1	
**5**	.487**	.356**	.372**	.575**	1

As shown in Table 7, correlation coefficients between *Smoothness Level* (1), *Scene Understanding* (2), *Visual Richness* (3), *Aesthetic Intention* (4), and *Visual Interest Degree* (5) were analyzed using *Spearman’s rank-order correlation*. All correlations were statistically significant at the 0.01 level (*two-tailed*), with coefficients such as 0.189** (1&2), 0.573** (1&4), and 0.575** (4&5). Specifically, the correlation coefficients between *Smoothness Level*, *Scene Understanding*, *Visual Richness*, *Aesthetic Intention,* and *Visual Interest Degree* were 0.490, 0.349, 0.368, and 0.491, respectively (all *p* < 0.05). These results indicate statistically significant associations between each dimension and *Visual Interest Degree*.

#### 3.3.4. Regression analysis.

Given that visual saliency is the central focus of this study, *Visual Interest Degree* was designated as the dependent variable. After confirming a statistically significant correlation between *Smoothness Level* and *Visual Interest Degree*, a regression analysis was conducted to examine the predictive relationship, with results presented in Table 8.

**Table 8:** Regression analysis of Visual Interest Degree and the other four items.

Model Summary (modle 1)							
Item	R	R^2^	Adj. R^2^	SE	DW	F	Sig.
SL	0.562	0.316	0.313	0.61381	1.941	160.798	<0.001
SU	0.372	0.138	0.136	0.69431	1.845	49.782	<0.001
VR	0.408	0.166	0.164	0.66785	1.887	66.967	<0.001
AI	0.708	0.501	0.498	0.59351	1.911	368.575	<0.001

Note: SL = Smoothness Level, SU = Scene Understanding, VR = Visual Richness, AI = Aesthetic Intention.

As shown in Table 8, a *Durbin-Watson* (DW) value of approximately 2 indicates no significant autocorrelation in the regression model, supporting the assumption of independent errors. With a coefficient of determination (R²) of 0.313, the model explains a meaningful proportion of variance in *Visual Interest Degree*, demonstrating acceptable explanatory power. The highly significant F-statistic (F = 160.798, p < 0.001) confirms the overall model’s statistical significance. As detailed in Table 9, *Smoothness Level* exhibits a positive and significant association with *Visual Interest* *Degree* (*t* = 10.162, *p* < 0.001). Collec*t*ively, these results indicate that *Smoothness Level* shows a strong positive association with visual interest (see Table 9).

**Table 9:** Regression coefficients and significance test for *Visual Interest Degree and* the other four indicators.

Variable	B	SE	β	t	Sig.
Smoothness Level	0.657	0.041	0.672	16.024	<0.001
Scene Understanding	0.301	0.036	0.358	8.361	<0.001
Visual Richness	0.368	0.047	0.358	7.830	<0.001
Aesthetic Intention	0.798	0.045	0.762	17.733	<0.001

*Note*: All models include a constant term with coefficients 1.382, 2.575, 2.404, 0.887, all statistically significant at p < 0.001.

Given the statistically significant correlation between *Scene Understanding* and *Visual Interest Degree*, a regression analysis was conducted to examine their relationship, with results presented in Tables 8 and 9. This approach enables an assessment of how variations in *Scene Understanding* relate to changes in *Visual Interest Degree*.

As shown in Table 8, a *Durbin-Watson* (DW) statistic of approximately 2 indicates no significant autocorrelation in residuals, supporting the assumption of independent model errors. An adjusted R² of 0.136 indicates that *Scene Understanding* explains 13.6% of the variance in *Visual Interest Degree*, reflecting moderate but meaningful explanatory power. The regression model is highly significant (F = 49.782, *p* < 0.001), reinforcing the reliability of the observed relationship. Table 9 presents the regression coefficients and significance tests for *Scene Understanding* and *Visual Interest Degree*. Significant *t-values* (*p* < 0.001) confirm a positive association, supporting the hypothesis that higher *Scene Understanding* is associated with increased visual interest.

Similarly, the aforementioned regression analysis method was applied to examine the relationships between *Visual Richness*, *Aesthetic Intention*, and *Visual Interest Degree*. Results showed all corresponding regression models were statistically significant, thereby supporting the hypothesized relationships within the experimentally designed scales (see Tables 8 and 9).

As shown in Table 9, the *t-value* and significance level (Sig.) for *Visual Richness* indicate a statistically significant positive effect on *Visual Interest Degree*. Tables 9 summarizes the regression results for *Aesthetic Intention* and *Visual Interest Degree*. Consistent with the preceding analyses, these models further confirm the statistical significance of the relationships, supporting the robustness of the observed effects.

#### 3.3.5. Statistical analysis of participant characteristics.

Verbal informed consent was obtained from all participants prior to their involvement in this study. The consent procedure was fully documented and witnessed by on-site researchers in accordance with ethical research standards. No minors were included in the participant pool.

The formal experiment involved 80 participants. A total of 400 questionnaires were collected across *Scene 1* and *Scene 2*. After excluding invalid responses (due to failed validation checks, extreme response times, or uniform answer patterns), 353 valid questionnaires were retained for *Scene 1* (response rate: 88.25%), including 68 for *Smoothness Level*, 73 for *Scene Understanding*, 71 for *Visual Richness*, 66 for *Aesthetic* *Intention*, and 75 for *Visual Interest Degree*. For *Scene 2*, 368 valid questionnaires were obtained (response rate: 92%), comprising 72 for *Smoothness Level*, 76 for *Scene Understanding*, 74 for *Visual Richness*, 71 for *Aesthetic Intention*, and 75 for *Visual Interest Degree*. Descriptive statistical analyses were conducted on the 353 valid samples from *Scene 1* and 368 valid samples from *Scene 2*. Participant demographic characteristics were summarized as follows: (1) *Gender*: 47 females (58.75%) and 33 males (41.25%). (2) *Age*: The majority of participants were between 19 and 31 years old, with 48 aged 19–23 (60%) and 32 aged over 23 (40%). (3) *Education level*: 53 participants held bachelor’s degrees (66.25%), 23 held master’s degrees (28.75%), and 4 held doctoral degrees (5%).

All participants majored in 3D animation design and possessed research experience in landscape animation and 3D modeling. As this study is rooted in a computational aesthetics framework focusing on landscape animation and virtual garden aesthetics, future work will extend to broader application domains and involve participants from more diverse academic backgrounds.

#### 3.3.6. Results and analysis of scale experiment.

This section presents an evaluation of a large-scale complex virtual environment (Test *Scene 2*), based on five perceptual and aesthetic scales: *Smoothness Level*, *Scene Understanding*, *Visual Richness*, *Aesthetic Intention*, and *Visual Interest Degree*. Each scale was assessed using five comparative methods: the proposed method, ACS, GA, GWO, and BAS. The results were systematically analyzed to evaluate performance differences, with detailed outcomes for the Smoothness Level dimension presented in Table 10.

**Table 10:** Comparative performance evaluation of five methods across evaluation scales.

Item	Method	M	SD	Median	Min	Max	p-value(vs. Proposed)	Sig. Marker
Smoothness Level	Proposed Method	6.45	0.978	6.5	5.2	7.0	―	―
	ACS	5.08	1.45	5.1	2.8	6.9	0.003	**
	GA	3.91	0.991	4.0	2.1	5.5	<0.001	**
	GWO	4.76	1.15	4.8	3.0	6.2	0.012	**
	BAS	3.82	0.967	3.8	2.0	5.3	<0.001	**
Scene Understanding	Proposed Method	5.87	1.10	6.0	3.5	7.0	―	―
	ACS	5.08	0.97	5.0	3.0	6.5	0.0004	**
	GA	5.13	0.85	5.0	3.2	6.8	0.0003	**
	GWO	4.53	0.81	4.5	2.8	6.0	<0.0001	**
	BAS	4.21	1.18	4.0	2.0	6.2	<0.0001	**
	Proposed Method	6.15	0.981	6.0	4.0	7.0	―	―
	ACS	4.12	1.212	4.0	2.0	6.5	<0.0001	**
Visual Richness	GA	4.51	0.867	4.5	2.5	6.8	<0.0001	**
	GWO	3.98	1.092	4.0	2.0	6.0	<0.0001	**
	BAS	3.24	0.925	3.0	1.5	5.5	<0.0001	**
Aesthetic Intention	Proposed Method	5.38	1.04	5.5	3.0	7.0	―	―
	ACS	4.24	0.99	4.0	2.0	6.5	<0.0001	**
	GA	4.58	1.25	4.5	2.5	6.8	0.0008	**
	GWO	3.89	0.87	4.0	2.0	6.0	<0.0001	**
	BAS	3.82	0.85	4.0	2.0	5.5	<0.0001	**
Visual Interest Degree	Proposed Method	6.43	1.08	6.5	4.0	7.0	―	―
	ACS	4.26	0.96	4.0	2.0	6.5	<0.0001	**
	GA	5.21	1.05	5.0	3.0	6.8	<0.0001	**
	GWO	3.87	0.83	4.0	2.0	6.0	<0.0001	**
	BAS	3.52	1.12	3.5	1.5	5.5	<0.0001	**

As shown in Table 10, the proposed method (M = 6.45, Median = 6.5, Min = 5.2, Max = 7.0) achieved high ratings on the *Smoothness Level scale* with moderate variability (SD = 0.978), reflecting robust overall performance and a response distribution consistent with a *7-point Likert scale*. ACS (M = 5.08, SD = 1.45) exhibited lower mean scores and greater dispersion, indicating less stable participant evaluations and weaker performance than the proposed method. GA (M = 3.91, SD = 0.991) scored near the neutral midpoint (4), suggesting average performance with no clear preference. GWO (M = 4.76, SD = 1.15) outperformed GA but remained substantially below the proposed method, demonstrating intermediate effectiveness. BAS (M = 3.82, SD = 0.967) yielded the lowest mean score, identifying it as the least effective approach. Statistical analysis indicates that the proposed method significantly outperformed all comparative methods (*p* < 0.01, **), providing strong evidence of superior performance and statistical significance.

On the *Scene Understanding scale*, the proposed method (M = 5.87, SD = 1.10) outperformed all comparison methods. ACS (M = 5.08, SD = 0.97) and GA (M = 5.13, SD = 0.85) showed moderate performance, with GA exhibiting slightly greater consistency. GWO (M = 4.53, SD = 1.15) performed below the proposed method, ACS, and GA. BAS (M = 4.21, SD = 1.18) had the lowest score and highest variability, indicating poor and inconsistent performance. The proposed method demonstrated superior accuracy and reliability in *Scene Understanding Scale*.

On the *Visual Richness scale*, the proposed method (M = 6.15) achieved the highest ratings, with results closely aligned with the expected *7-point Likert distribution*. ACS (M = 4.12, SD = 1.212) exhibited lower performance and high variability, indicating inconsistent evaluations. GA (M = 4.51, SD = 0.867) outperformed ACS with more consistent ratings. GWO (M = 3.98, SD = 1.092) scored below the proposed method, ACS, and GA, reflecting limited visual richness. BAS (M = 3.24, SD = 1.18) had the lowest score and high dispersion, confirming poor and unreliable performance. The proposed method clearly surpassed all others in visual richness and consistency.

As listed in Table 10, the proposed method (M = 5.38, Median = 5.5, Min = 3.0, Max = 7.0) achieved the highest score on the *Aesthetic Intention scale*, indicating strong capacity to elicit the intended aesthetic perceptions. ACS (M = 4.24, SD = 0.99) obtained lower scores with moderate variability, suggesting less consistent participant evaluations. GA (M = 4.58, SD = 1.25) outperformed ACS but remained below the proposed method, with greater rating dispersion reflecting higher inter-subject variability. GWO (M = 3.89, Median = 4.0, Min = 2.0, Max = 6.0) yielded the lowest mean score, underperforming all other methods and indicating limited effectiveness in conveying aesthetic intent. The results demonstrate that the proposed method significantly surpasses comparative approaches in aesthetic intention.

As shown in Table 10, the proposed method (M = 6.43, Median = 6.5, Min = 4.0, Max = 7.0) achieved the highest score on the *Visual Interest Degree scale* with moderate dispersion (SD = 1.08), consistent with a *7-point Likert scale*. ACS (M = 4.26, SD = 0.96) scored lower with higher variability, indicating less stable responses. GA (M = 5.21, SD = 1.05) performed better than ACS but remained below the proposed method, with comparable rating consistency. GWO (M = 3.87, SD = 0.83) scored lower than the proposed method, ACS, and GA, with less variation, suggesting more uniform but less favorable ratings. BAS (M = 3.52, SD = 1.12) had the lowest scores and highest variability, reflecting inconsistent perceptions. Significant differences were observed across methods (*p* < 0.01, ***), with *post-hoc* tests showing that the proposed method consistently outperformed all other approaches across the five scales: *Smoothness Level*, *Scene Understanding*, *Visual Richness*, *Aesthetic Intention*, and *Visual Interest Degree*. These results indicate superior effectiveness in eliciting the intended visual experience.

### 3.4. Analysis of aesthetic scoring

**Fig 8:**
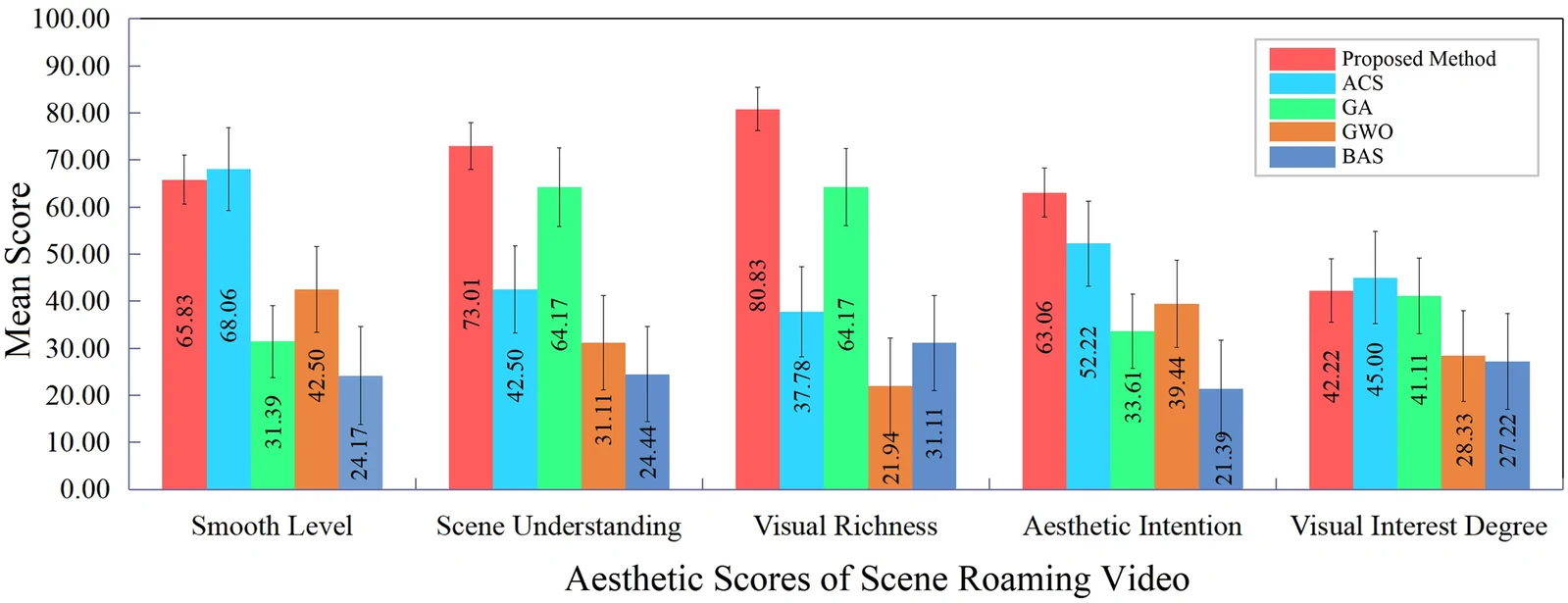
Aesthetic scores of path roaming generated by different methods in *Scene 1.*

During the experiment, participants virtually toured paths generated by different methods in *Scene 1* and rated the aesthetic qualities of landscape sequences from various camera viewpoints. To facilitate standardized comparative analysis across scenarios, all participant scores were normalized to a 100-point scale, and averages were computed. As noted earlier, the rating scale comprised five criteria: *Smoothness Level*, *Scene Understanding*, *Visual Richness*, *Aesthetic Intention*, and *Visual Interest Degree*. Results are presented in Fig 8. For *Smoothness Level*, both the proposed method and ACS received high scores, significantly outperforming GA, GWO, and BAS. Although the proposed method scored slightly lower than ACS on *Smoothness Level*, it achieved substantially higher scores on the remaining four criteria compared with the other methods.

The proposed method scored slightly below ACS in *Smoothness Level* (reduced by 3.28%)—a difference that can be regarded as negligible, yet outperformed GA, GWO, and BAS by 109.72%, 54.89%, and 172.36%, respectively, demonstrating superior visual smoothness. In *Scene Understanding*, it achieved improvements of 71.79%, 13.78%, 134.68%, and 198.73% over ACS, GA, GWO, and BAS, indicating stronger spatial and contextual coherence. For *Visual Richness*, mean scores increased by 113.95%, 25.96%, 268.42%, and 160.08% relative to the comparative methods, reflecting richer visual integration and greater compositional diversity. In *Aesthetic Intention*, gains of 20.76%, 87.62%, 59.89%, and 194.81% further indicate better alignment with artistic goals. On *Visual Interest Degree*, despite a slight decrease compared to ACS (reduced by 6.18%), the proposed method exceeded GA, GWO, and BAS by 2.70%, 49.03%, and 55.11%, respectively, confirming its broad robustness. Overall, the results demonstrate the consistent superiority of the proposed method across all aesthetic dimensions.

**Fig 9:**
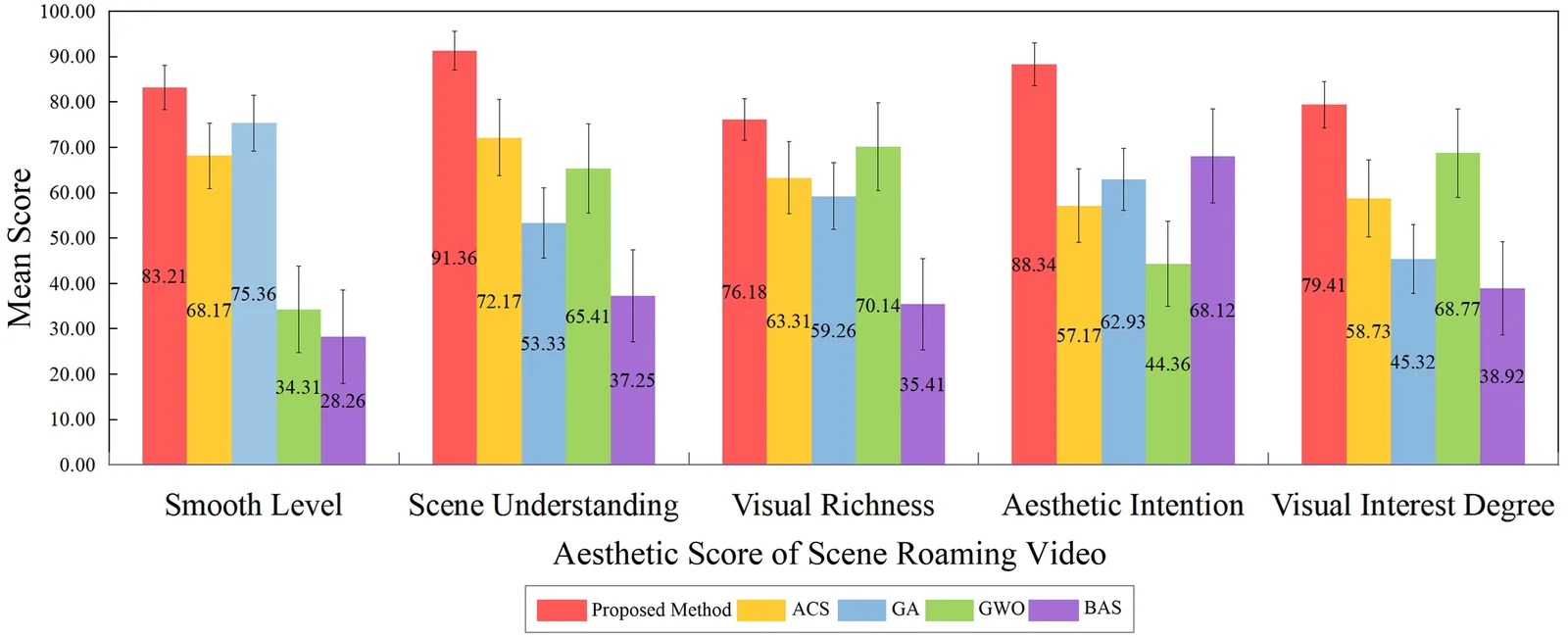
Aesthetic scores of path roaming generated by different methods in *Scene 2.*

In the more complex and large-scale Scene 2, the same questionnaire used in Scene 1 was adopted (see Fig 9). The proposed method consistently outperforms ACS, GA, GWO, and BAS across all evaluation dimensions. Specifically, the performance improvements reach 22.06%, 10.42%, 142.52%, and 194.44% in *Smoothness Level*; 26.59%, 71.31%, 39.67%, and 145.26% in *Scene Understanding*; 20.33%, 28.55%, 8.61%, and 115.14% in *Visual Richness*; 54.52%, 40.38%, 99.14%, and 29.68% in *Aesthetic Intention*; and 35.21%, 75.22%, 15.47%, and 104.03% in *Visual Interest Degree*. These results validate the robustness and overall superiority of the proposed method for producing high-quality visual outputs in complex scenarios.

Initial experiments were carried out in structured virtual environments, including heritage parks and ancient architectural complexes. To further verify the applicability of the proposed method in more diverse and challenging scenarios such as urban landscapes and complex natural terrains, we extended the experimental analysis to a large-scale digital twin city to validate its generalizability.

Fig 1(H)–(M) presents the reconstructed landscape of *Handan New Century Square* in the digital twin project, which comprises 45 large-scale buildings and corresponding urban spatial nodes. The virtual building cluster is constructed by precisely replicating the geometric proportions of real-world counterparts. As shown in Fig 1(H)–(I), the left subfigure displays real urban landmarks, whereas the right subfigure shows the corresponding digital twin scene, which faithfully reproduces the visual perception and immersive experience of real urban navigation.

Fig 1(J)–(L) depicts a partial view of the digitally reconstructed urban environment. The integration of diverse modern architectural styles within the virtual scene poses substantial challenges to visual perception guidance and aesthetic feature extraction, further demonstrating the complexity of constructing perceptually consistent and visually attractive digital urban landscapes.

**Fig 10:**
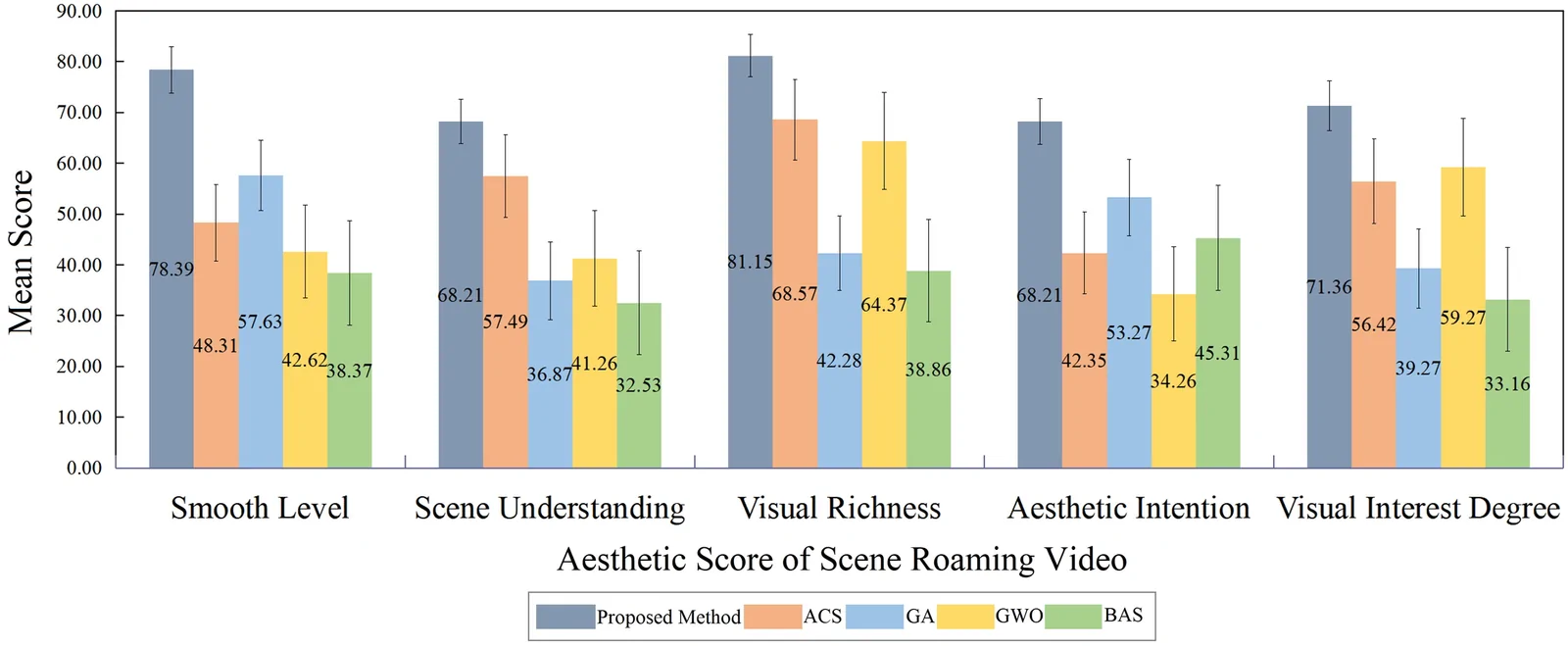
Aesthetic evaluation scores of digital urban roaming in *Handan New Century Square.*

Five approaches, including the proposed method, ACS, GA, GWO, and BAS, are employed for virtual camera path planning in the digital twin city. The generated roaming videos are systematically evaluated in terms of aesthetic quality under a unified framework, covering five core dimensions: smoothness, scene understanding, visual richness, aesthetic intention, and visual interest. As shown in Fig 10, the proposed method achieves optimal performance across all evaluation criteria, which verifies its capability to generate visually pleasant and cognitively consistent camera roaming paths.

The proposed method achieves higher scores than ACS, GA, GWO, and BAS in all aesthetic evaluation dimensions of scene roaming videos, including *Smoothness Level*, *Scene Understanding*, *Visual Richness*, *Aesthetic Intention*, and *Visual Interest Degree*, thereby verifying its superior overall performance. Relevant detailed analyses are presented in the following sections.

For *Smoothness Level*, the mean score reached 78.39, compared with 57.63 for the best-performing baseline method (GA), representing a 36.02% improvement. The proposed method outperformed ACS by 62.26%, GWO by 83.93%, and BAS by 104.30%, exceeding GA by nearly 20.76 points and confirming smoother path generation. For *Scene Understanding*, the score reached 68.21, surpassing ACS (57.49) by 10.72 points (+18.65%). Improvements over GA, GWO, and BAS were 85.00%, 65.32%, and 109.68%, respectively, indicating stronger spatial and contextual clarity. In terms of Visual Richness, the score reached 81.15, exceeding ACS (68.57) by 12.58 points (+18.35%), with further gains of 91.93% (GA), 26.07% (GWO), and 108.83% (BAS), reflecting richer visual content. For *Aesthetic Intention*, the score reached 68.21, compared with 53.27 for GA, representing a 14.94-point improvement. Relative gains over ACS, GA, GWO, and BAS were 61.06%, 28.05%, 99.09%, and 50.54%, respectively, indicating improved alignment with aesthetic design goals. On *Visual Interest Degree*, the proposed method achieved a score of 71.36, exceeding GWO (59.27) by 12.09 points. Performance gains over ACS, GA, GWO, and BAS were 26.48%, 81.72%, 20.40%, and 115.20%, respectively, demonstrating stronger viewer engagement.

Overall, the proposed method consistently outperformed all baseline methods across all five evaluated dimensions, with performance improvements ranging from 18% to over 115%. These results demonstrate its effectiveness in enhancing scene understanding, aesthetic richness, and visual engagement in complex environments.

**Fig 11:**
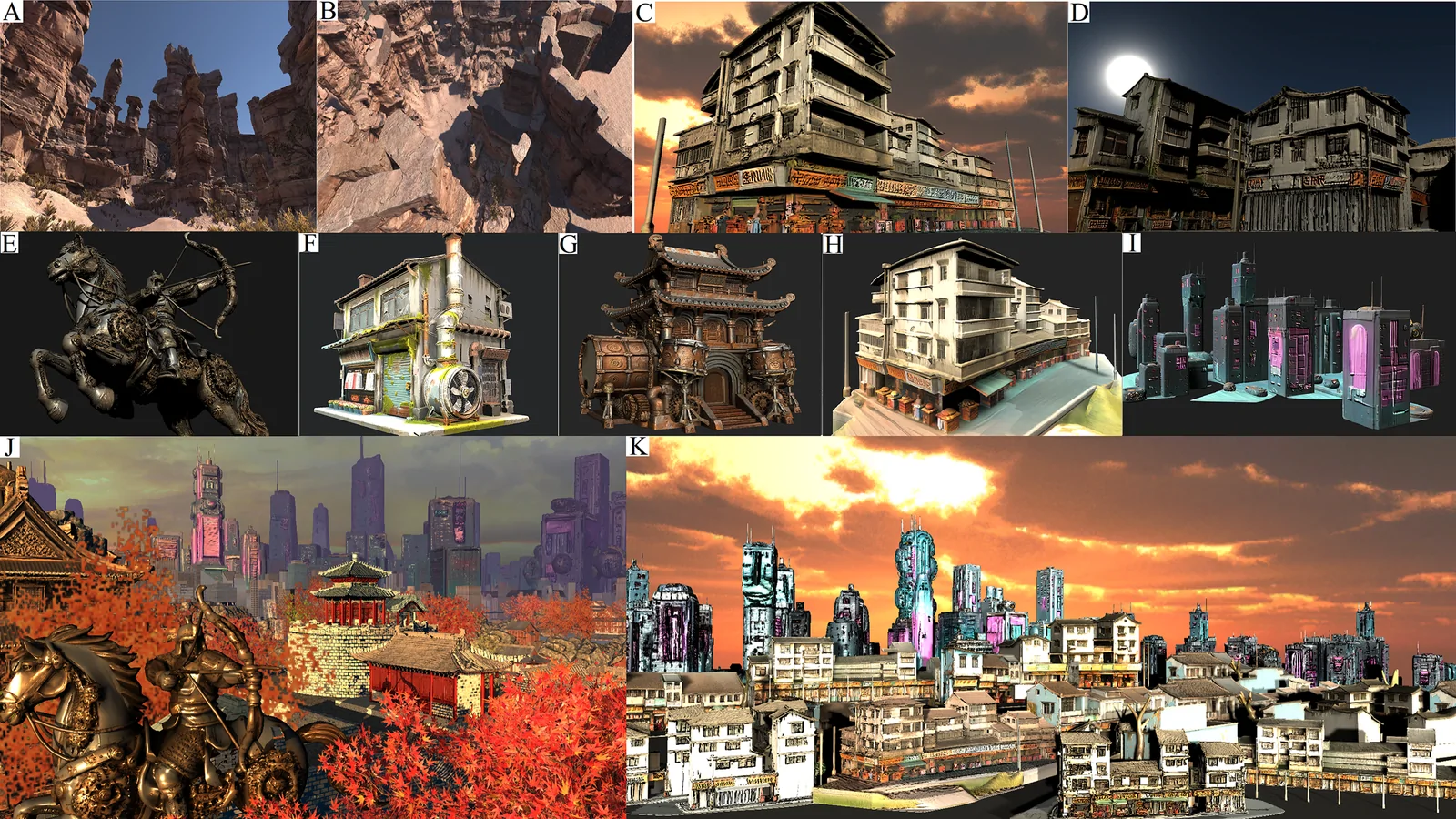
Constructed and procedurally generated 3D scenes. (A)–(B) Complex Danxia landform scenes; (C)–(I) Text-to-3D generated monolithic architectural assets from sci-fi narratives; (J) Cyberpunk-style ancient architecture; (K) Magical realist urban village scenes from original literary works.

To assess the performance of the proposed method under more complex and irregular natural terrain conditions, we constructed a *Danxia* landform within the game environment, featuring numerous boulders with dense pores and cracks, interconnected by highly tortuous and heterogeneous traversable pathways. A systematic comparison was conducted between the proposed method and baseline algorithms (ACS, GA, GWO, BAS) across all key aesthetic dimensions. The results demonstrate that the proposed method not only maintains competitive performance in such challenging environments but also achieves superior aesthetic quality, evidenced by higher mean scores across multiple evaluation criteria, thereby confirming its generalization ability and robustness, as shown in Fig 11(A)–(B).

**Fig 12:**
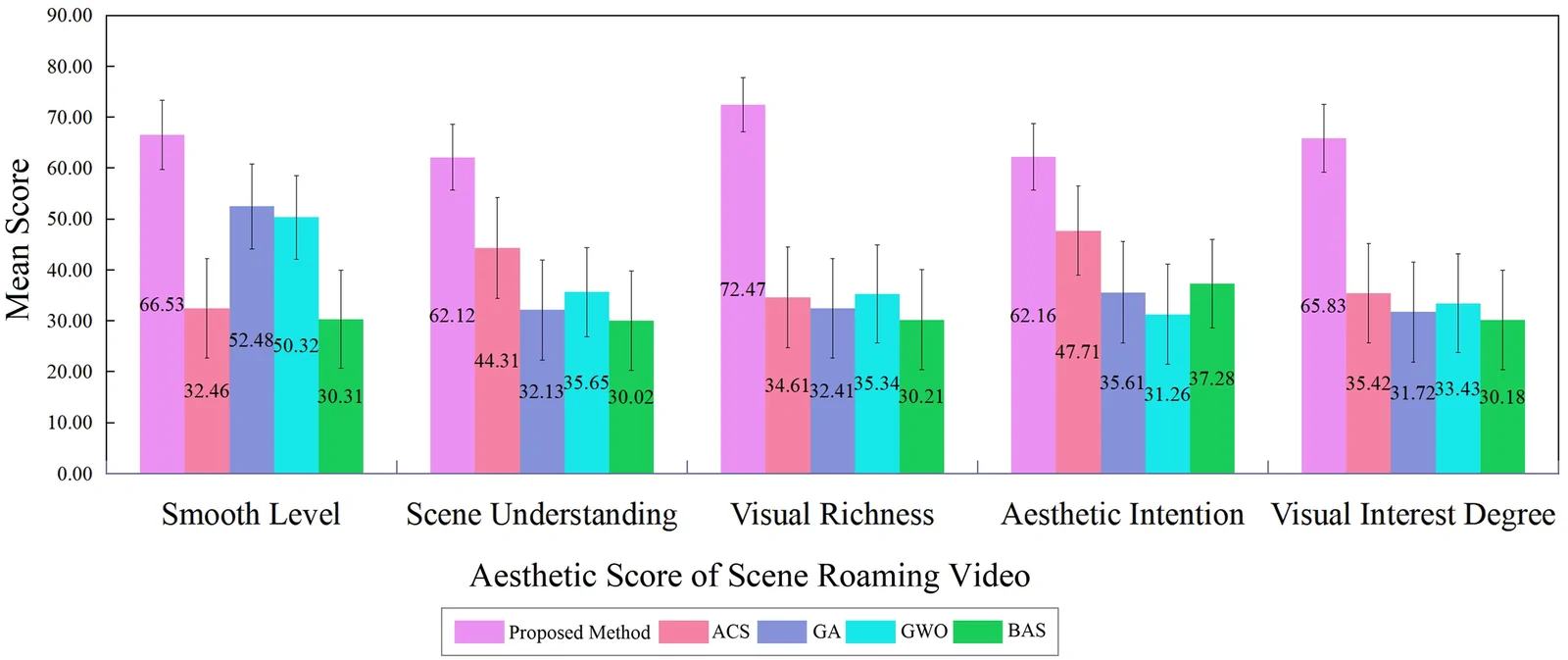
Aesthetic scores for irregular rock layer landform roaming.

Fig 12 illustrates the superiority of the proposed method. The mean aesthetic scores across dimensions reveal that it outperforms all other methods (ACS, GA, GWO, BAS) in *Smoothness Level*, *Scene Understanding*, *Visual Richness*, *Aesthetic Intention*, and *Visual Interest Degree*, with evident performance advantages. It achieves the highest scores in all five core dimensions. Compared with ACS, GA, GWO, and BAS, performance improvements are substantial: *Smoothness Level* (+104.96%, + 26.77%, + 32.21%, + 119.50%), *Scene Understanding* (+39.97%, + 93.34%, + 74.25%, + 106.93%), *Visual Richness* (+109.39%, + 123.60%, + 105.06%, + 139.89%), *Aesthetic Intention* (+30.29%, + 74.56%, + 98.85%, + 66.74%), and *Visual Interest Degree* (+85.85%, + 107.53%, + 96.92%, + 118.12%).

For example, in *Visual Richness*, the proposed method scores 72.47, far exceeding GWO (35.34), ACS (34.61), GA (32.41), and BAS (30.21). Similar trends are observed in two key evaluation metrics: *Smoothness Level*, in which the proposed method achieves a score of 66.53 compared to 52.48 for GA, and *Scene Understanding*, in which it attains 62.12 compared to 44.31 for ACS. Even when compared with ACS, one of the strongest baseline methods, the proposed method shows substantial improvements, scoring 14.45 points higher in *Aesthetic Intention* and 17.81 points higher in *Scene Understanding*. In contrast, GA, GWO, and BAS consistently score lower, with relatively small differences among them but a pronounced gap relative to the proposed method. On *Smoothness Level*, for instance, their scores are 52.48, 50.32, and 30.31, respectively, all well below the proposed method’s 66.53.

**Fig 13:**
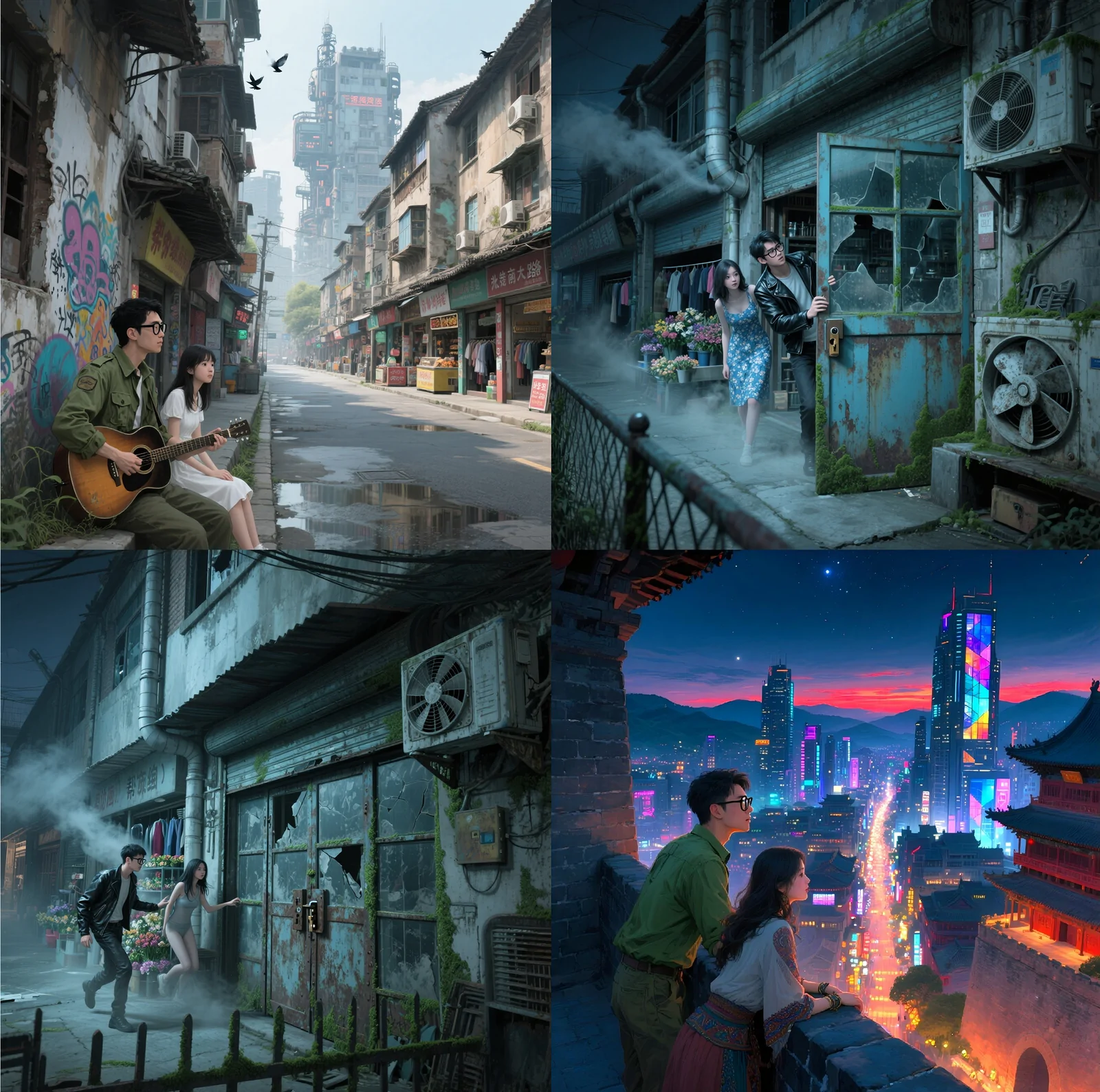
AIGC-generated 2D scene concept visualizations derived from the original literary work.

To further expand the applicability of our method in interdisciplinary fields, we utilized the officially published science fiction film and television scripts from the art fund project led by one of the authors of this paper as experimental materials [74,75]. An innovative approach was adopted to explore the intelligent generation and rapid construction of VR film and television scenes (see Fig 13).

In the specific implementation, the research team, based on this science fiction literary work (https://doi.org/10.5281/zenodo.18058055), utilized the Text-to-3D capability of large-scale AI models to generate VR science fiction scenes integrating magical realism and cyberpunk styles. These scenes served as experimental prototypes to validate the applicability of the proposed method, as shown in Fig 11(C)–(I).

To support reproducible research and open scientific dissemination, we have publicly released all structured prompt templates, prompt examples, and parameter conversion tables for VR scene generation on the Zenodo open-access platform. These materials carry the following persistent DOIs: https://doi.org/10.5281/zenodo.19467233 and https://doi.org/10.5281/zenodo.19905900. These open and well-documented resources enable full replication of our semantic parsing, parameter mapping, and end-to-end text-to-3D generation pipeline, without dependence on proprietary 3D assets or copyrighted literary materials. All 69 buildings in this complex scene were generated exclusively by the *Tencent Hunyuan 3D* large-scale AI model based on the textual descriptions from the science fiction film and television script (without any post-hoc manual processing of the 3D models), and their aesthetic layout was optimized using a genetic algorithm, as illustrated in Fig 11(J)–(K).

In the intelligent generation of VR scenes, we employed natural language processing (NLP) techniques: the original text was segmented via a text fragment-label mapping strategy, in which each text fragment was mapped to a four-dimensional label system. For example, the text fragment “*a fallen city shrouded in a blood-red sunset glow*; *a mechanical geisha stalking the alleys of a dilapidated urban village*; *swallows darting through cobweb-like interwoven cables*; *a desolate, mournful floodplain covered with massive sandstone and flint resembling skeletal remains; and flame-red sand willow forests stretching across the wilderness*” corresponds to the following labels [75]: geographical characteristics (fallen city, dilapidated urban village, floodplain landform), cyberpunk elements (mechanical geisha, cable network), magical imagery (blood-red sunset imagery, flame-red sprawling sand willow forests), and narrative functions (lyrical scene, sorrowful atmosphere).We optimized the layout of the generated complex scene using an interactive genetic algorithm to enhance visual appeal and aesthetic intent. Meanwhile, using saliency maps, aesthetic rules, and narrative logic as constraints, we generated a VR scene layout that balances aesthetic quality, attention guidance, and narrative integrity. This layout faithfully restores the literary imagery and aesthetic experience of the original work to a certain extent, while also conveying the absurdity and atmosphere of the original work—a fusion of soft science fiction in a wasteland punk style and magical realism.

**Fig 14:**
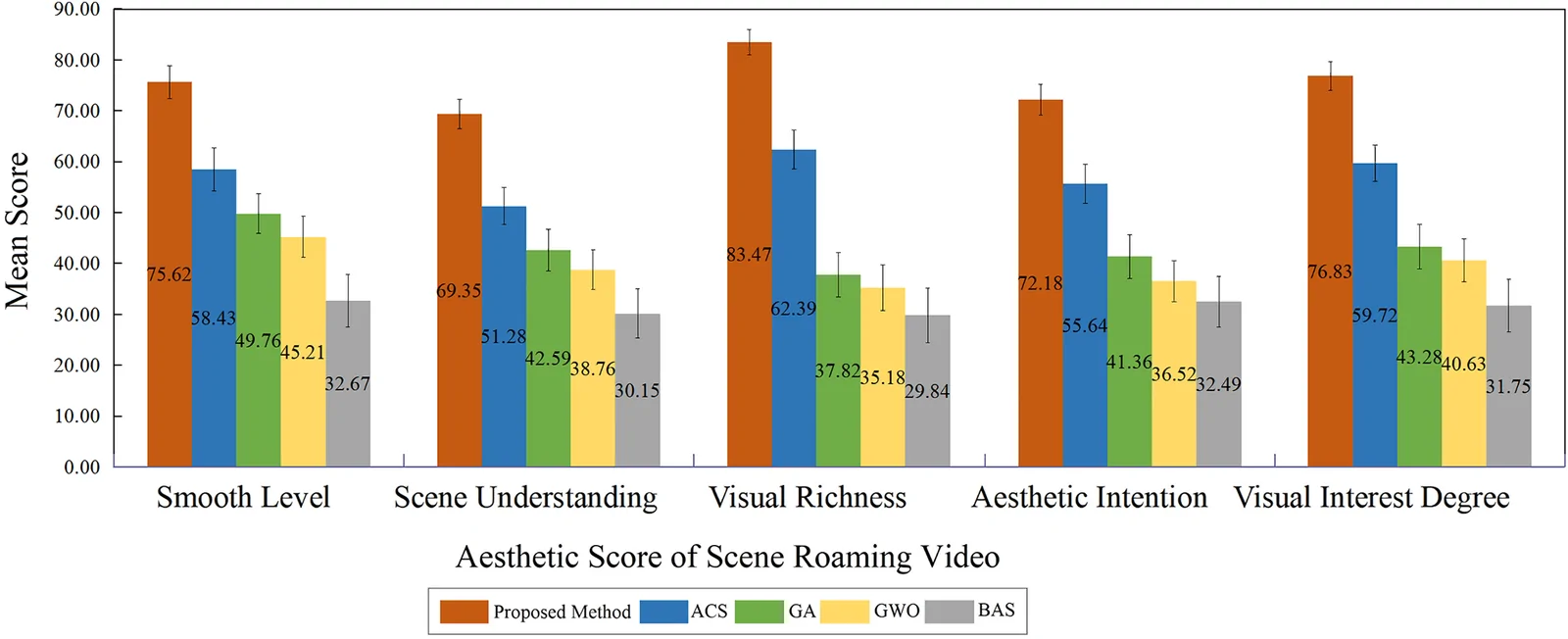
Aesthetic evaluation scores of various methods for *Text-to-3D* scene generation (Mean ± Standard Deviation).

In the comparative experiments based on *Text-to-3D* scene generation, we adopted the same five aesthetic evaluation metrics as defined in the preceding sections (i.e., *Smoothness Level*, *Scene Understanding*, *Visual Richness*, *Aesthetic Intention*, and *Visual Interest Degree*). Five comparative algorithms were employed, including the proposed method, ACS, GA, GWO, and BAS. A double-blind scoring process was conducted by 20 students majoring in digital media art with practical experience in 3D scene design. All scores were standardized to a 100-point scale, followed by calculation of mean values and standard deviations. The experimental results are presented in Fig 14.

*Text-to-3D* scenes usually embody specific artistic creation intentions, yet comparative algorithms mostly rely on fixed path generation rules and thus struggle to align with the aesthetic features of the scenes. In contrast, the proposed method introduces a visual *perception-guided mechanism*, enabling the generated paths to closely match the artistic tonality of the scenes, thereby achieving more favorable performance.

In the test environment of *Text-to-3D* scene generation characterized by high randomness, complexity, and semantic particularity, the proposed method maintained remarkably stable performance advantages across all five aesthetic evaluation dimensions, with the average improvement ranging from 28.65% to 179.76%. This result demonstrates the strong generalization capability of the proposed method: its core mechanism is not only applicable to structured virtual environments (e.g., heritage parks, digital twin cities) and irregular natural terrains (e.g., *Danxia* landform) but also effectively adapts to the semantic ambiguity and visual diversity of *Text-to-3D* scenes. Through dynamic path optimization, in-depth semantic parsing, and precise matching of aesthetic intentions, the proposed method achieves a comprehensive enhancement of the roaming experience, providing an efficient solution for intelligent roaming path planning in *Text-to-3D* scenes.

To intuitively reveal disparities in subjective aesthetic indicators of the five algorithms under five test scenarios, we aggregate dimensional scores into a consolidated summary table (see Table 11).

**Table 11:** Comprehensive Summary of Subjective Aesthetic Scores of All Algorithms across Five Test Scenes.

Scene	Algorithm	SL	SU	VR	AI	VD
Scene 1 (Basic Test)	Proposed Method	65.83	**73.01**	**80.83**	**63.06**	42.22
	ACS	**68.06**	42.50	37.78	52.22	**45.00**
	GA	31.39	64.17	64.17	33.61	41.11
	GWO	42.50	31.11	21.94	39.44	28.33
	BAS	24.17	24.44	31.11	21.39	27.22
Scene 2 (Natural Landform)	Proposed Method	**83.21**	**91.36**	76.18	**88.34**	**79.41**
	ACS	68.17	72.17	63.31	57.17	58.73
	GA	75.36	53.33	59.26	62.93	45.32
	GWO	34.31	65.41	**70.14**	44.36	68.77
	BAS	28.26	37.25	35.41	**68.12**	38.92
Scene 3 (Digital Twin City)	Proposed Method	**78.39**	**68.21**	**81.15**	**68.21**	**71.36**
	ACS	48.31	57.49	68.57	42.35	56.42
	GA	57.63	36.87	42.28	53.27	39.27
	GWO	42.62	41.26	64.37	34.26	59.27
	BAS	38.37	32.53	38.86	45.31	33.16
Scene 4 (Irregular Rock Landform)	Proposed Method	**66.53**	**62.12**	**72.47**	**62.16**	**65.83**
	ACS	32.46	44.31	34.61	47.71	35.42
	GA	52.48	32.13	32.41	35.61	31.72
	GWO	50.32	35.65	35.34	31.26	33.43
	BAS	30.31	30.02	30.21	37.28	30.18
Scene 5 (AIGC Text-to-3D)	Proposed Method	**75.62**	**69.35**	**83.47**	**72.18**	**76.83**
	ACS	58.43	51.28	62.39	55.64	59.72
	GA	49.76	42.59	37.82	41.36	43.28
	GWO	45.21	38.76	35.18	36.52	40.63
	BAS	32.67	30.15	29.84	32.49	31.75

In conclusion, the proposed method achieves a consistent and significant advantage across all aesthetic evaluation dimensions, demonstrating superior performance in path planning and intelligent guidance for enhancing landscape roaming aesthetics.

## 4. Discussion

This study initially validates the proposed method in classical garden heritage digital twin scenes using real-world geometric datasets, and subsequently extends the approach to AI-generated 3D scenes from text prompts. The unified theoretical framework based on visual saliency and aesthetic guidance achieves consistent performance in both real-scene geometric reconstruction and text-driven 3D generation, effectively bridging traditional cultural heritage digitization and modern AIGC virtual scene creation. Despite its contributions, this study has several limitations:

(1) The sample lacks demographic diversity, consisting predominantly of female participants aged 19–23 majoring in 3D animation programs. While this composition aligned with the thesis’s thematic focus, it limits the generalizability of the findings—a constraint further shaped by the scope of the underlying research project. Future work should incorporate participants from more diverse disciplines and backgrounds to comprehensively examine how domain expertise, cognitive traits, and individual differences influence engagement in interactive virtual environments.
(2) The research primarily focuses on classical garden landscapes and commercial district digital twins—environments characterized by relatively simple spatial configurations that facilitate visual saliency modeling. More complex scenarios, such as those involving intricate terrains, multi-layered architectures, and dynamic changes, as well as interactions with virtual characters (which can modulate scene cognition), remain insufficiently explored. As virtual applications continue to evolve, future research should adopt more integrative methodologies [64,65,70,71,73,76–79]: including the analysis of context-dependent aesthetic features, the development of adaptive guidance systems based on virtual cameras and digital humans, and the evaluation of how different exploration paradigms influence users’ emotional and cognitive responses. In addition, extending the algorithm to support dynamic environments with real-time updates to visual regions and obstacles represents a promising direction for future research. Such efforts should further examine the impact of large-scale and complex virtual environments on usability, depth of aesthetic appreciation, and sustained user engagement. These investigations should be supported by rigorous validation across diverse contexts (e.g., urban landscapes, natural ecosystems, and multi-level spatial environments) to strengthen methodological robustness.
(3) This work concentrates on refining and validating classical optimization algorithms, situating it within the foundational stage of *aesthetic-driven* scene exploration research. Owing to constraints related to sample size, hardware resources, funding, and time, future studies will transition toward deep learning-based intelligent path planning approaches. This progression will involve constructing standardized datasets, defining quantifiable evaluation metrics, and establishing reproducible benchmarks, thereby forming a scalable research framework.

## 5. Conclusion

This study presents a visual perception-guided virtual camera path planning framework integrating mesh saliency detection, improved DAPF, and enhanced ACO. It prioritizes visually salient and aesthetically prominent regions in large-scale complex virtual scenes, resolving the classic trade-off between optimization efficiency and aesthetic quality in conventional path planning methods.

Compared with four baseline algorithms (ACS, GA, GWO, BAS), the proposed method achieves superior performance in path length (32.09% reduction), exploration efficiency (43.39% time reduction), and visual saliency accumulation. Four typical scenarios—heritage digital twins, urban landscapes, natural terrains, and AIGC-generated scenes—further verify the efficacy of the presented method.

Subjective user evaluation validates that the framework outperforms baselines in motion smoothness, scene comprehension, visual richness, aesthetic expressiveness and visual appeal. Statistical analysis reveals significant differences at *p* < 0.01 in all evaluation dimensions against baseline algorithms.

The proposed framework exhibits broad applicability in heritage conservation, virtual cultural tourism, 3D digital twin navigation, and text-to-3D scene roaming. Powered by the integration of saliency detection and intelligent optimization, it achieves strong generalization across structured, unstructured, and AI-generated complex scenes.

Four future research directions are highlighted, including extending the framework to dynamic virtual scenes, integrating deep learning into path planning pipelines, recruiting diverse participants to alleviate sample homogeneity bias, and validating the method in multi-layer architectural and interactive virtual human scenarios.
